# Fabrication and Validation of an Economical, Programmable, Dual-Channel, Electronic Cigarette Aerosol Generator

**DOI:** 10.3390/ijerph182413190

**Published:** 2021-12-14

**Authors:** Dominic L. Palazzolo, Jordan Caudill, James Baron, Kevin Cooper

**Affiliations:** 1 Department of Physiology, DeBusk College of Osteopathic Medicine, Lincoln Memorial University, Harrogate, TN 37752, USA; jordan.caudill@lmunet.edu (J.C.); james.baron@lmunet.edu (J.B.); 2 Department of Chemistry and Physics, School of Mathematics & Sciences, Lincoln Memorial University, Harrogate, TN 37752, USA; Kevin.Cooper@lmunet.edu

**Keywords:** vaping, ECIG aerosol generator, validation process, atomizer temperatures, electrical parameters, aerosol particle distribution, nicotine recovery

## Abstract

Vaping (inhalation of electronic cigarette-generated aerosol) is a public health concern. Due to recent spikes in adolescent use of electronic cigarettes (ECIGs) and vaping-induced illnesses, demand for scientific inquiry into the physiological effects of electronic cigarette (ECIG) aerosol has increased. For such studies, standardized and consistent aerosol production is required. Many labs generate aerosol by manually activating peristaltic pumps and ECIG devices simultaneously in a predefined manner. The tedium involved with this process (large puff number over time) and risk of error in keeping with puff topography (puff number, duration, interval) are less than optimal. Furthermore, excess puffing on an ECIG device results in battery depletion, reducing aerosol production, and ultimately, its chemical and physical nature. While commercial vaping machines are available, the cost of these machines is prohibitive to many labs. For these reasons, an economical and programmable ECIG aerosol generator, capable of generating aerosol from two atomizers simultaneously, was fabricated, and subsequently validated. Validation determinants include measurements of atomizer temperatures (inside and outside), electrical parameters (current, resistance and power) of the circuitry, aerosol particle distribution (particle counts and mass concentrations) and aerosol delivery (indexed by nicotine recovery), all during stressed conditions of four puffs/minute for 75 min (i.e., 300 puffs). Validation results indicate that the ECIG aerosol generator is better suited for experiments involving ≤100 puffs. Over 100 puffs, the amount of variation in the parameters measured tends to increase. Variations between channels are generally higher than variations within a channel. Despite significant variations in temperatures, electrical parameters, and aerosol particle distributions, both within and between channels, aerosol delivery remains remarkably stable for up to 300 puffs, yielding over 25% nicotine recovery for both channels. In conclusion, this programmable, dual-channel ECIG aerosol generator is not only affordable, but also allows the user to control puff topography and eliminate battery drain of ECIG devices. Consequently, this aerosol generator is valid, reliable, economical, capable of using a variety of E-liquids and amenable for use in a vast number of studies investigating the effects of ECIG-generated aerosol while utilizing a multitude of puffing regimens in a standardized manner.

## 1. Introduction

Electronic cigarettes (ECIG), originally intended as a harm-reduction alternative to traditional smoking, typically contain an atomizer tank for containment of ECIG-liquid (E-liquid) and a battery supplying power to vaporize the E-liquid [1]. Voltage from the ECIG’s battery is applied to the resistance coil (located within the atomizer tank), resulting in heat transfer to the E-liquid, causing it to vaporize, thus generating the aerosol. Inhalation of ECIG-generated aerosol (i.e., vaping) has fast become a serious public health concern, especially among adolescents and young adults [2]. This concern, amid recent vaping-induced illnesses, with and without illicit substances present in the E-liquid [3,4,5,6], demands there be more scientific inquiry into the physiologic effects of ECIG-generated aerosol. Consequently, a means of generating consistent aerosol under CORESTA standards [7] (3 s puffs every 30 s with puff volume of 55 mL) is essential. Commercial vaping machines capable of generating aerosol with consistency are available [8,9,10], but expensive. As an alternative, many ECIG investigators generate aerosol by utilizing equipment (e.g., pumps, flow meters, data loggers, and timers) and consumable supplies (tubing and connectors, Pasteur pipettes, etc.) ordinarily found in the laboratory [11,12], but these improvised “in-house” setups are often less then optimal in generating a uniform aerosol on a per puff basis, especially when voltage is applied to the system using the ECIG’s rechargeable battery, which is subject to depletion.

The generation of uniform aerosol using an “in house” vaping machine is dependent on three factors. First, simultaneous activation and inactivation of the equipment is required. For example, if the peristaltic pump (used to transport aerosol) and the ECIG battery (the power source used to generate the aerosol) are not synchronized, the volume of aerosol generated will vary from puff to puff. This is especially true when the pump and power source are actuated manually [12,13,14]. With manual activation and inactivation of pump and power source there is the additional risk of inaccurately executing puff topography (i.e., puff number, duration, interval) due to human error. Secondly, the aerosol airflow must remain constant from puff to puff. This means that the pump flow rate needs to be calibrated to the desired airflow. Finally, the electrical parameters (voltage, current, resistance and power) of the ECIG device must remain constant. Voltage is usually supplied to the ECIG device by a lithium-ion rechargeable battery. As the battery drains from continued puffing on the ECIG device, voltage, current, and, consequently, power will all decrease, which effects aerosol production and ultimately the chemical and physical nature of the aerosol [15,16,17,18]. Furthermore, as lithium-ion batteries age, their ability to store charge is reduced, thus limiting the output voltage and power [19]. The maximum resistance in the ECIG device is determined by the material and length and thickness of the wire used to construct the coil within the atomizer. Coils of varying resistances can be purchased and installed within the atomizer to suit the vaping preference of the user [20]. Providing that the applied voltage and the resistance of the coil both remain constant, the rate at which electrical energy is converted to heat (i.e., power) remains constant, resulting in uniform aerosol production.

With this goal of consistently producing uniform aerosol with every puff, we fabricated an inexpensive dual-channel ECIG aerosol generator capable of generating aerosol from two different sources simultaneously. This ECIG aerosol generator can actuate the pumps and power source synchronously. The device is programmable, consequently reducing risk of human error in keeping with puff topography. Finally, the rechargeable lithium-ion batteries are replaced with a constant voltage power supply, thus eliminating the effects of battery drain and aging. In this report, the fabrication of the ECIG aerosol generator and its incorporation into a system capable of transporting aerosol to where it is desired (i.e., exposure chamber or bubbled into media) is detailed. Furthermore, we validate the use of this ECIG aerosol generator by monitoring its thermal and electrical properties, measuring particle distribution and mass concentration of the aerosol output, and determining aerosol delivery using nicotine as a dosimetry marker. These determinants were all collected while stressing the system with 300 continuous puffs at a rate of four puffs per minute. We have thus constructed a device that is economical, valid, and capable of delivering reliable standardized doses of aerosol on a per puff basis.

## 2. Materials and Methods

### 2.1. Reagents and Supplies

Laboratory materials and reagents were obtained from Fisher Scientific (Waltham, MA, USA) unless otherwise noted.

### 2.2. E-Liquid and Ecig Atomizer

The E-liquid was composed of 50% propylene glycol and 50% vegetable glycerine (aka glycerol) with 20 mg/mL of 99% (S)-(-)-nicotine (Alfa Aesar, Tewksbury, MA, USA). No flavorings were added. The nicotine concentration per cigarette equivalent is higher than the typical concentration of nicotine in a tobacco cigarette but comparable to the high-end nicotine concentration found in a number of commercially available E-liquids [21]. The E-liquid was aerosolized using a newly constructed programmable dual-channel aerosol generator (see details below) providing a constant voltage of 3.8 V (in parallel) to two TFV16 SMOK^®^ (Shenzhen IVPS technologies Co. LTD, Shenzhen, China) atomizers with glass tanks capable of housing 9 mL of E-liquid. The atomizers come preinstalled with a TFV16 Mesh 0.17 Ω mesh nichrome coils for a targeted power output of ~60 W per coil. When operated, the vents to the atomizers were kept wide open. A new coil was used with every 300-puff experiment.

### 2.3. Fabrication of the Ecig Aerosol Generator

**Figure 1:**
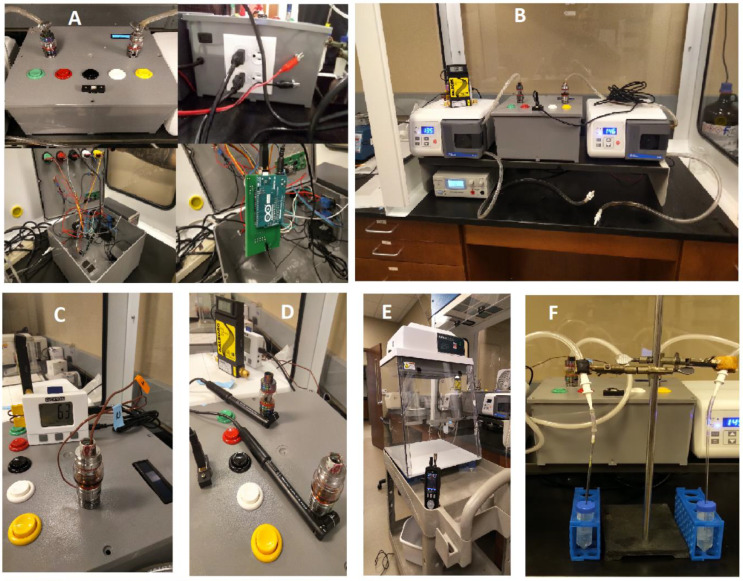
Programmable, dual-channel ECIG aerosol generator. (**A**) Housing for the electrical components of the programmable, dual-channel ECIG aerosol generator. (**B**) ECIG aerosol generator as part of the complete system, which includes two peristaltic pumps and a DC voltage power supply. (**C**) Measurement of temperature inside the tank (orange label) and outside the tank (blue label) using K-type thermocouples. (**D**) Measurement of magnetic field using magnetic field sensors. (**E**) Measurement of particle counts (0.3, 0.5, 1.0, 2.5, 5.0 and 10 µm size) and mass concentration of particles (2.5 and 10 µm size). (**F**) Aerosol trapping for the measurement nicotine recovery.

The programmable dual-channel ECIG aerosol generator with fixed DC voltage was inexpensively fabricated using components listed in Supplemental Table S1. In Figure 1A, various views of the ECIG aerosol generator housing (12” × 12” × 6” plastic junction box) are shown. Figure 1A top left photo, shows the working surface of the generator, displaying two electrically parallel SMOK^®^ atomizers, each attached to 510 connectors, five color coded push buttons, (green = starts system as programmed by computer code, red = aborts system program, black = activates atomizers and pumps for as long as the button is depressed, white = activates atomizers only, and yellow = activates pumps only), USB connector to upload control code and a two line LCD interfaced with an Arduino UNO Rev. 3 controller board (Arduino, Somerville, MA, USA) (Supplemental Figure S1). These components are connected to an in-house designed circuit board which was printed by JLCPCB (Shenzhen, Guangdong, China) (Supplemental Figure S2) to the specifications shown in the Supplemental Figure S3 schematic.

The back of the ECIG aerosol generator is depicted in Figure 1A top right photo and shows a rocker switch to power the generator. Additionally shown are the terminals connecting the atomizers of the ECIG aerosol generator to the variable DC voltage switching power supply (Tekpower, Montclair, CA, USA) via red and black 14 AGW wires. The variable DC voltage power supply allows the user to achieve a desired power output based on the coil resistance as well as operate the device in a single atomizer configuration. Two of the four 120 V receptacles serve as outlets for two Fisherbrand GP1000 peristaltic pumps (Waltham, MA, USA) and a 120 V inlet for main power. Figure 1A bottom left photo shows the exposed circuitry inside the ECIG aerosol generator, and Figure 1A bottom right photo shows a closeup of the Arduino and the printed circuit board. All control circuitry is fitted inside the plastic junction box. Figure 1B is a photo of the complete system; peristaltic pumps flank the ECIG aerosol generator on both sides and the variable DC voltage supply is located under the peristaltic pump on the left side. Located on top of the left peristaltic pump is an Aalborg GFM flow meter (Orangeburg, NY, USA) used to equilibrate aerosol flow rate.

The C++ code used to program the ECIG aerosol generator is shown in Supplemental File S1. It allows for the number of puffs, puff duration and puff interval to be altered by the investigator as desired. The computer application screen is shown in Supplemental Figure S4. The boot time for initiation of the GP1000 peristatic pumps is 4.1 s and is subtracted from the puff interval. As an aside, the boot time remains constant so long as the GP1000 pumps are used. The boot time for other peristaltic pumps will differ and can be accommodated by altering a variable in the code.

### 2.4. Puff Topography

Two Fisherbrand GP1000 peristaltic pumps (Waltham, MA, USA), one for each atomizer, were used to transport the generated aerosol through ~60 inches of Saint Gobain Tygon S3 (B-44-4X) Precision Tubing (ID = 8.0 mm, 1.6 mm wall thickness). Before each run, pump flow rates were equilibrated to 1.1 L/min (or 55 mL/puff) using an Aalborg GFM flow meter to simulate the flow of air intake during a 3 s puff by the ECIG aerosol generator. The puffing protocol consisted of up to 300 cycles (75 min) of a 3 s puff followed by a 12 s rest period. While puff duration and flowrate followed Cooperation Centre for Scientific Research Relative to Tobacco (CORESTA) Guidelines of Method No. 81 [7], we elected to use a puff interval of 12 s (four puff cycles per minute) rather than 27 s (2 puff cycles per minute). Doubling the number of puff cycles from 2 to 4 per minute allowed for all ECIG aerosol generator tests to be validated under more stressful conditions to ensure the heat generated during extensive puffing regimens would not damage the electrical components of the aerosol generator. It should also be pointed out that CORESTA guidelines represents an average puff topography of most individuals and that doubling the number of puffs from two to four per minute is not beyond the scope of all who vape. Consequently, the experimental conditions used in this study (in terms of exposure to aerosol particles of various sizes and nicotine delivery) could translate to individuals who vape excessively. To this end, for one experiment in which the electrical parameters of the ECIG aerosol generator were tested, puff duration was increased to five seconds while the puff interval was decreased to five seconds (i.e., six puff cycles per minute) for a total of five puffs.

All puffing experiments were conducted within a P20 Purair ductless fume hood (Airscience, Fort Meyers, FL, USA) with a high-efficiency particulate air (HEPA) filter.

### 2.5. Measurement of Atomizer Temperatures

Temperatures inside and outside the atomizer were determined using a Dickenson (Addison, IL, USA) SM325 data logger equipped with two flexible K-type-thermocouple temperature probes (Figure 1C). One probe was inserted down the barrel of the atomizer as close to the coil as possible, the other probe was attached to the outer edge of the atomizer just below the glass tank. Temperatures were monitored over a period of 300 puffs (or 75 min) on the ECIG generator. The sampling rate was 12 temperature readings per minute for both probes and were collected using Dickson’s SW03 software (Addison, IL, USA).

### 2.6. Measurement of Atomizer Magnetic Fields and Currents

**Figure 2:**
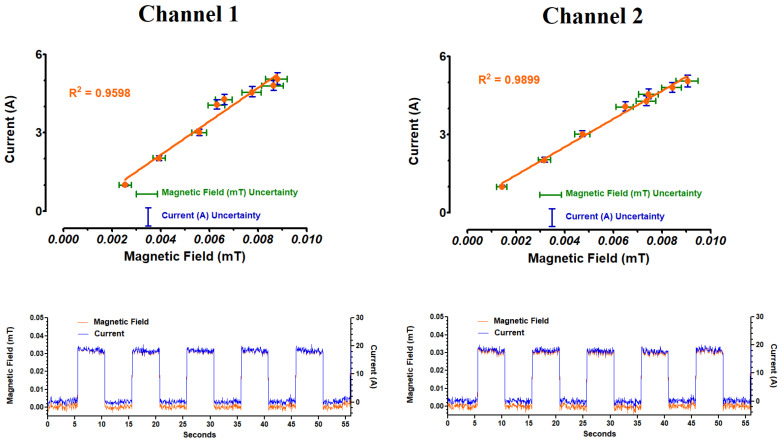
Relationship between magnetic field (mT) and current (A). Upper panels illustrate the linearity of the calibration curves for both channels, and the bottom panels show the actual magnetic fields and currents established from the first five puffs of the ECIG aerosol generator (five-second puffs with a five-second interval between puffs) for both channels.

The magnetic fields generated by the current present in the atomizer coils when activated were measured using Vernier (Beaverton, OR, USA) magnetic field sensors (Figure 1D). The sensor signals were sent to a Vernier LabPro data acquisition interface (Beaverton, OR, USA) and stored for later analysis. Magnetic field data were collected over a period of five puffs at the start of puffing (five-second puffs, ECIG aerosol generator active; with a five-second interval between puffs, ECIG aerosol generator inactive) or over a period of five puffs after 100, 200 and 300 puffs continuously (three-second puffs, ECIG aerosol generator active; with a twelve-second interval between puffs, ECIG aerosol generator inactive). The sampling rate was four magnetic field readings per second. Providing that the environment and geometry were kept consistent throughout the measurements, the generated magnetic field is linearly proportional to the current with the constant of proportionality determined by the structure of the coil as well as the geometry and materials present in the system. Figure 2 depicts this linear relationship for both channels running simultaneously.

### 2.7. Measurement of Particle Size Distributions and Mass Concentrations Produced by the Ecig Aerosol Generator

Counts (particles/ft^3^) for 0.3, 0.5, 1.0, 5.0 and 10 µm particle sizes and mass concentrations (µg/m^3^) were determined using a portable PCE-PCO 2 particle counter (PCE Americas, Jupiter, FL). The particle counter was set to count particle distributions and mass concentrations for 50 s per 2.31 L flow, with a start delay of five seconds, and a five-second interval between cycles (i.e., one minute/cycle). In the first experiment, particle counts, and mass concentrations were determined for 75 cycles (75 min), which corresponded to 300 continuous puffs (75 min) of the ECIG aerosol generator. In the second experiment, particle counts, and mass concentrations were determined for 15 cycles without any aerosol generation (baseline), followed by three sets of 15 cycles in which aerosol was generated for 12 puffs (three minutes) at the beginning of each counting set. The aerosol generated was delivered into an AirClean 600 (AirClean^®^ Systems, Creedmoor, NC, USA) Vertical Laminar Flow Fume Hood through a side entry port at the rear base of the right side of the hood. The dimensions of the flow hood (24” × 24” × 30”) served as a semi-confined space in which aerosol could accumulate but was unrestrained. During aerosol delivery, the filter on the top of the hood was removed and the front door panel remained half open. The PCE-PCO 2 counter was placed one meter, directly diagonal from the aerosol entry point and the aerosol that wafted to the particle counter was analyzed. During particle counting the fans on the AirClean 600 and the P20 Purair ductless fume hood were shut off. A depiction of the particle counting setup is shown in Figure 1E.

### 2.8. Aerosol Trapping in Growth Media

The ECIG aerosol generator delivered 0, 100, 200 or 300 puffs of aerosol (from E-liquid containing 20 mg/mL nicotine) directly into brain heart infusion (BHI) broth, a common bacterial growth media used in our laboratory. The outlet tubing of the peristaltic pumps was downsized with a small piece of tubing which fits the top ends of a 1 mL serologic pipettes. The serologic pipets passed through bored holes into closed but vented 50 mL conical tubes (i.e., homemade impinger) as described in Nelson et al. [22], thus allowing for a fraction of the aerosol to be trapped in 10 mL of BHI growth media. Figure 1F illustrates this configuration.

### 2.9. High Performance Liquid Chromatography (HPLC) of Nicotine

**Figure 3:**
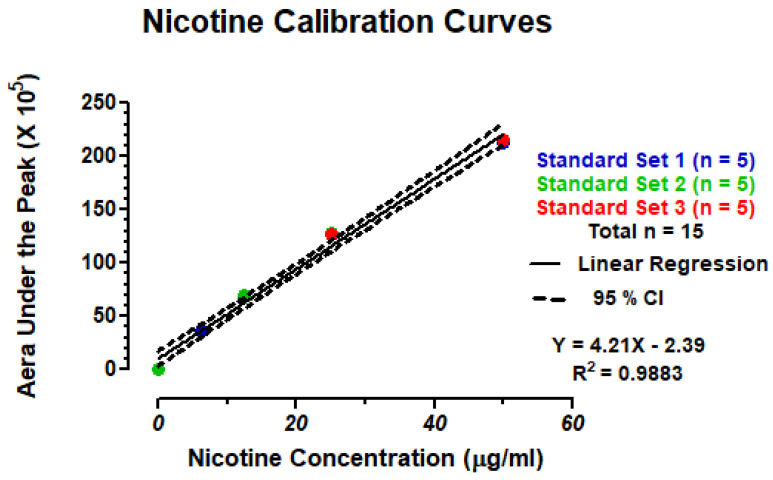
Nicotine calibration curves.

Standard solutions of 99% (S)-(-)-nicotine were prepared in BHI broth at concentrations of 6.25, 12.5, 25 and 50 µg/mL. Standards and fourfold dilution of BHI samples exposed to 0, 100, 200 or 300 puffs of ECIG aerosol containing nicotine were analyzed by HPLC coupled with photodiode array detection as previously described [21,22,23]. A Shimadzu HPLC system (Columbia, MD, USA) was used to quantitate nicotine and included the following: a photodiode array detector (SPD-M20A), dual pumps (LC-20AT), a column oven (CTO-20A), an in-line membrane degasser (DGU-20A3R) and a Rheodyne 7725I manual injector with a 20 µL loop (40 µL injection volume). Nicotine was separated on a Phenomenex (Torrance, CA, USA) 15-cm, Kinetex^®^ 5 µm reversed-phase C-18 column preceded by a Phenomenex Security Guard. The column temperature was maintained at 35 °C. Nicotine was detected at ultraviolet (UV) wavelengths between 230 and 300 nm, and quantifications were carried out at 260 nm. The mobile phase was delivered at a rate of 1 mL/minute in gradient fashion where mobile phase A consisted of 10% acetonitrile in 20 mM ammonium formate adjusted to pH 8.5 with 50% ammonium hydroxide and mobile phase B consisted of 100% acetonitrile. Mobile phase A decreased from 100% to 80% from 0 to 10 min and from 80% to 20% from 10 to 20 min and increased from 20% to 100% from 20 to 21 min and remained at 100% until the end of the run time at 30 min. Mobile phase B increased from 0% to 20% from 0 to 10 min and from 20% to 80% from 10 to 20 min and decreased from 80% to 0% from 20 to 21 min and remained at 0% until the end of the run time at 30 min. Nicotine elutes at a retention time of 10.5 min and the nicotine standard curve, shown in Figure 3, is linear (R^2^ = 0.9883). Chromatographic parameters were PC-controlled using a Shimadzu Lab Solutions workstation (Columbia, MD, USA).

### 2.10. Statistical Analysis

All data are expressed as the means ± standard error of the means (SEM) and within- and between-channel comparisons are made using a one-way ANOVA followed by Tukey’s multiple comparison test. Significance is achieved when *p* < 0.05. Version 5 of Prism (GraphPad Software, San Diego, CA, USA) was used to perform all statistical calculations.

## 3. Results

### 3.1. Temperature Profiles

**Figure 4:**
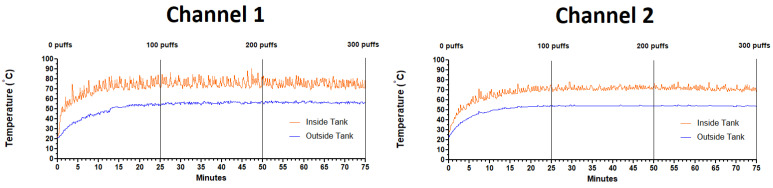
Temperature profiles (orange = inside temperature and blue = outside temperature) for two E-liquid atomizers running simultaneously on two channels for 75 min (or 300 puffs).

As shown in Figure 4, the temperatures inside and outside the atomizers (both channels) climb from room temperature at the start of puffing and reach a plateau between 15 and 20 min of puffing. Once plateau is achieved, the temperatures remain stable for the duration of the puffing regimen (i.e., 75 min or 300 puffs total). The means ± SEM for inside and outside temperatures of each atomizer were calculated for the first, second and third sets of 100 puffs (i.e., 25 min intervals) and are shown in Table 1. The temperatures (both inside and out) for the first set of 100 puffs are significantly lower than the second and third sets, but not between the second and third sets of 100 puffs. Temperatures (both inside and out) for the first, second and third sets of 100 puffs in channel 1 were significantly different from the first, second and third sets of 100 puffs in channel 2, respectively. Differences in the inside temperatures were 5.6, 4.2 and 3.8 °C for the first, second and third sets of 100 puffs, respectively, while differences in the outside temperatures were 1.7, 1.9 and 2.5 °C for the first, second and third sets of 100 puffs, respectively.

### 3.2. Electrical Parameters (the First Five Puffs)

**Figure 5:**
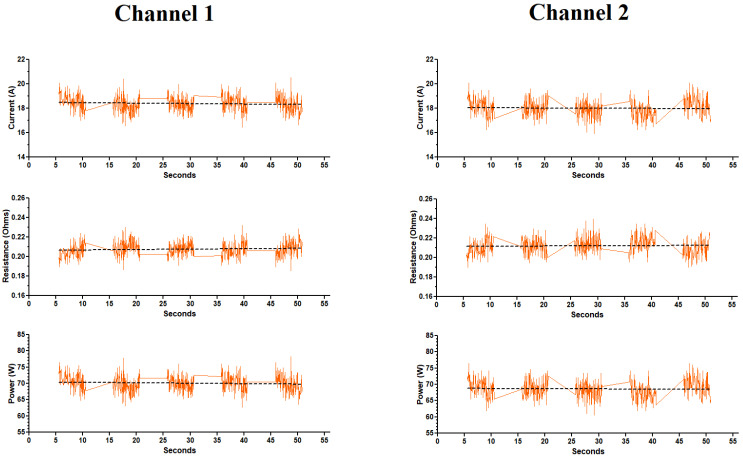
Electrical parameter profiles during the first five puffs of the ECIG aerosol generator running both channels simultaneously. Dashed line represents the trend line for each parameter.

The electrical parameters (i.e., current, resistance and power) after the first five puffs (or 55 s) produced by the ECIG generator, at both channels, are exhibited in Figure 5, revealing stability within the individual channels; however, there is a statistical difference in current, resistance and power between channels 1 and 2, as shown in Table 2. Furthermore, the calculated resistance deviates from the stated resistance on the coil by 0.04 Ω. Using a small commercially available multimeter, a direct measure of the coil’s resistance was 0.24 Ω (or 0.12 Ω across the two channels in parallel), which differs from the stated resistance on the coils by 0.07 Ω.

### 3.3. Electrical Parameters (300 Continous Puffs)

**Figure 6:**
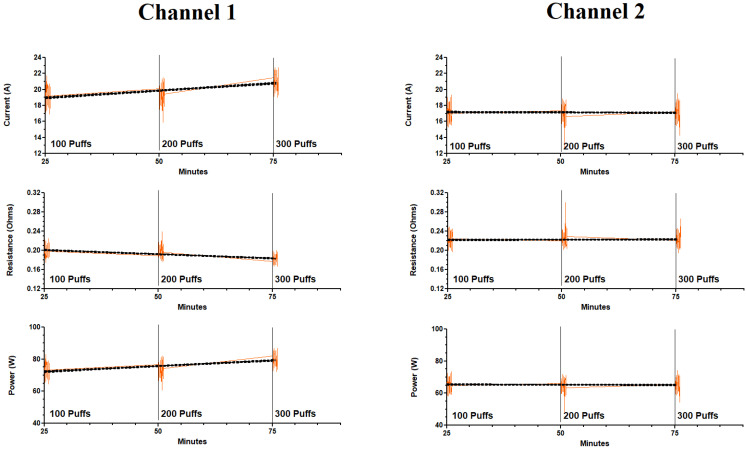
Electrical parameter profiles during 300 continuous puffs of the ECIG aerosol generator running both channels simultaneously. Dashed line represents the trend line for each parameter.

Figure 6 illustrates the effects of 300 continuous puffs over a 75 min period on current, resistance and power while the ECIG generator is running both channels simultaneously. The current, resistance and power were determined over a five-puff interval commencing after the first 100 puffs, then again after 200 puffs and 300 puffs. Three hundred continuous puffs resulted in an overall increase in amperage in channel 1 but remained stable in channel 2. Subsequently, calculations of resistance and power reflected the values obtained from the currents. From Table 3A, the current, resistance and power in channel 1 ranged from 19.2 to 21.1 A, 0.181 to 0.197 Ω and 73.3 to 80.3 W, respectively. In channel 2, the current, resistance and power ranged from 17.1 to 17.2 A, 0.221 to 0.224 Ω and 65.0 to 65.7 W, respectively. In addition, values or current, resistance and power obtained from channel 1 after 100, 200 300 puffs were statistically different from values obtained from channel 2 after 100, 200, and 300 puffs. A comparison of the average current, resistance, and power after the first five puffs (from Figure 5) and after 100 puffs (from Figure 6), for both channels 1 and 2, is shown in Table 3B revealing both within-channel and between-channel differences.

### 3.4. Particle Size Distributions and Mass Concentrations

**Figure 7:**
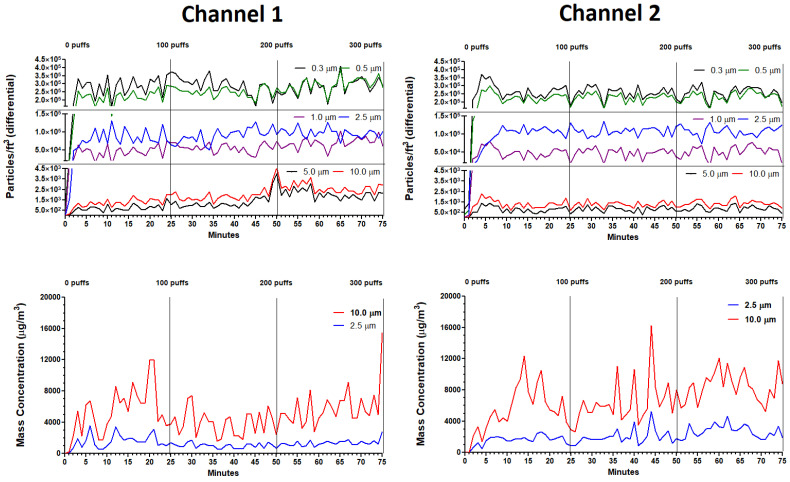
Particle size (**upper panels**) and mass concentration profiles (**lower panels**) during 300 continuous puffs of the ECIG aerosol generator running both channels simultaneously.

EXPERIMENT 1 (300 Continuous Puffs): Profiles for particle size distributions and mass concentrations during 300 continuous puffs (for both channels) are shown in Figure 7 and the associated statistics are described in Table 4. The profiles for the 0.3 and 0.5 µm particle sizes ranged from 1.5 × 10^5^ to 4.0 × 10^5^ particles/ft^3^in both channels with the 0.3 µm particles having slightly higher counts than 0.5 µm particles. The profiles for the 1.0 and 2.5 µm particle sizes ranged from 4.5 × 10^4^ to 1.5 × 10^5^ particles/ft^3^ in both channels with the 2.5 µm particles having slightly higher counts than 1.0 µm particles. The profiles for the 5.0 and 10 µm particle sizes ranged from 0 to 4.5 × 10^3^ particles/ft^3^ in both channels with the 10 µm particles having slightly higher counts than 5.0 µm particles. The mass concentration profiles for the 2.5 and 10 µm particle sizes ranged between 1000 and 9000 µg/m^3^ in both channels with the 10 µm particles having slightly higher concentrations than 2.5 µm particles. Statistically (Table 4), the variability over 300 puffs within channels is higher for channel 1 than for channel 2, and the variability between channels increases over time (i.e., with increasing puff number).

**Figure 8:**
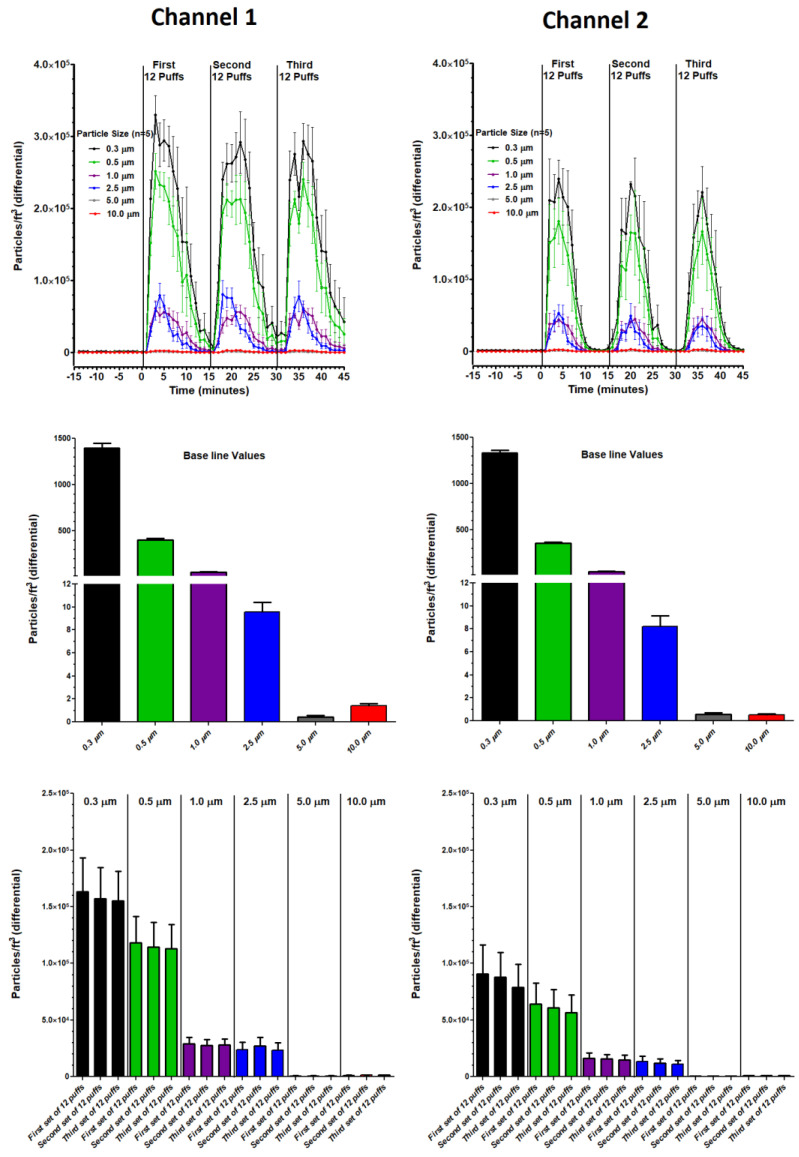
Profiles of particle size counts (**upper panels**), average baseline particle size counts (**middle panels**) and average particle size counts for each of three sets of twelve puffs (**lower panels**) of the ECIG aerosol generator running both channels simultaneously. Each bar represents the mean ± SEM (n = 15).

**Figure 9:**
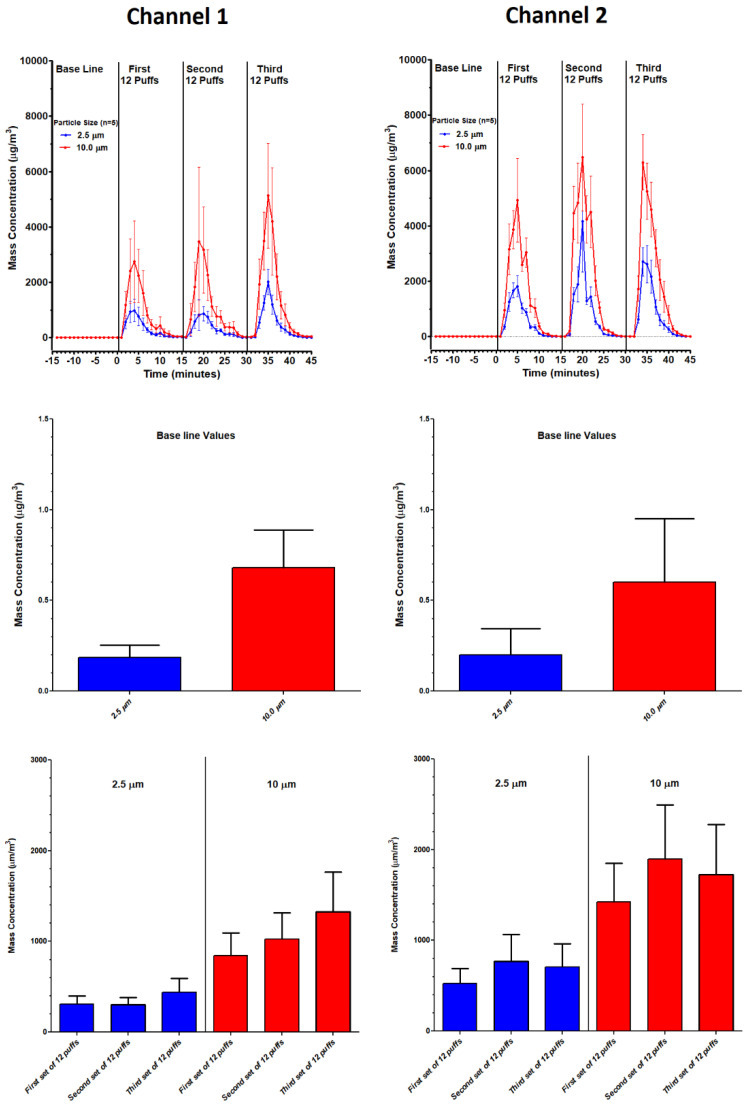
Profiles of mass concentrations (**upper panels**), average baseline mass concentrations (**middle panels**) and average mass concentrations for each of three sets of twelve puffs (**lower panels**) of the ECIG aerosol generator running both channels simultaneously. Each bar represents the mean ± SEM (n = 15).

EXPERIMENT 2 (Three Sets of Twelve Puffs): Profiles for particle size distributions and mass concentrations over three sets of twelve puffs (for both channels) are shown in Figure 8 and Figure 9, respectively. The associated statistics are described in Table 4. In Figure 8, each set of twelve puffs (for both channels) elevated all particle counts to peak levels within 5 to 7 min from the start of puffing and returned to baseline within 15 min. Average particle counts during baseline (−15 to 0 min), set 1 (0 to 15 min), set 2 (15 to 30 min) and set 3 (30 to 45 min) were consistent and no significant variations within channels and between channels were observed in any of these profiles (Table 4). Similarly, in Figure 9, each set of twelve puffs (for both channels) elevated all mass concentrations to peak levels within 5 to 7 min from the start of puffing and returned to baseline within 15 min. Average mass concentrations during baseline, set 1, set 2 and set 3 were consistent and no significant variations within and between channels were observed in any of these profiles (Table 4).

### 3.5. E-Liquid Vaporization

**Figure 10:**
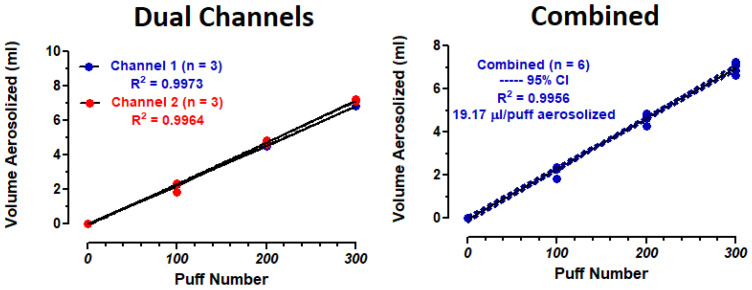
The volume of E-liquid aerosolized on a per puff basis using the ECIG aerosol generator.

The volume of E-liquid aerosolized after 100, 200 and 300 puffs and on a per puff basis is shown in Table 5 and in Figure 10. The amount of E-liquid aerosolized after 0, 100, 200 and 300 puffs of the ECIG aerosol generator forms a linear relationship with R^2^ values of 0.9973, 0.9964 and 0.9956 for channels 1, 2 and channels 1 and 2 combined, respectively.

### 3.6. Aerosol Delivery Based on Nicotine Recovery

**Figure 11:**
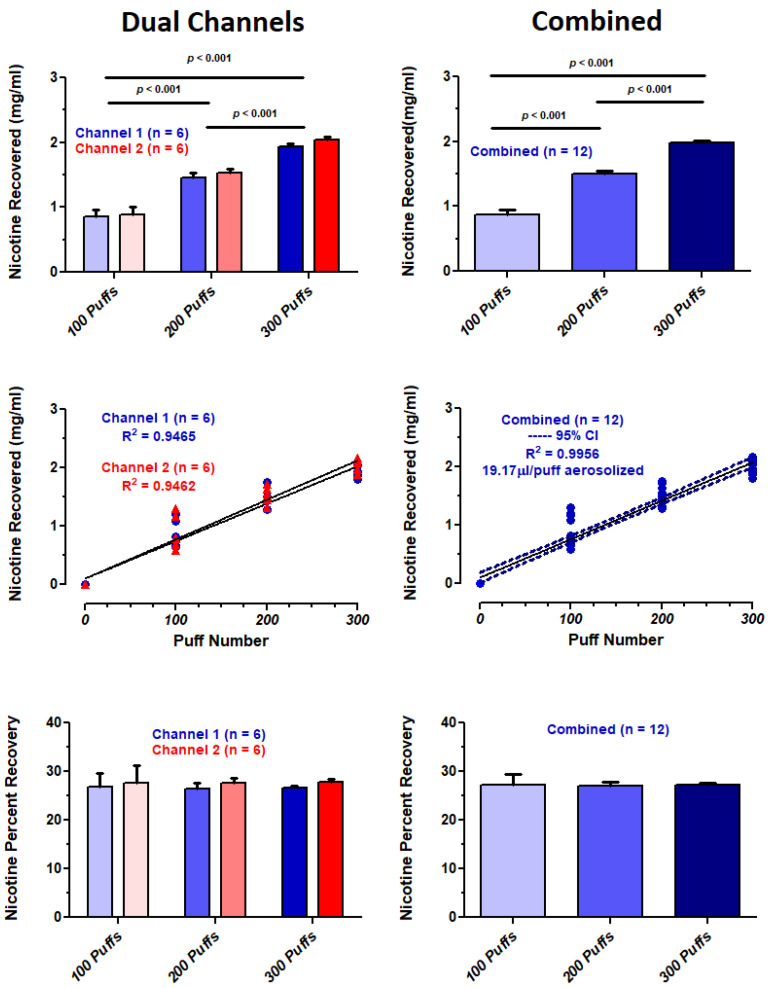
The amount of nicotine recovered after 0, 100, 200 and 300 puffs, as well as the percent recovery of nicotine, using the dual-channel ECIG aerosol generator. The amount of nicotine (mg/mL) recovered as determined by HPLC is multiplied by a dilution factor of twenty. Percent recoveries were determined by dividing the nicotine recovered (values in the middle panels) by the theoretical maximum concentration of nicotine delivered in either 0, 100, 200 or 300 puffs. The theoretical maximum amount of nicotine in 0, 100, 200 or 300 puffs is based on the amount of aerosolized E-liquid (19.17 µL/puff) which is added to the initial 10 mL volume of BHI, thus giving final volumes of 10, 11.92, 13.83 or 15.75 mL, respectively. Since the E-liquid has a nicotine concentration of 20 mg/mL, the theoretical maximum concentrations of nicotine in BHI are 0, 3.22, 5.54 and 7.30 mg/mL for 0, 100, 200 and 300 puffs, respectively. Each bar represents the mean ± SEM.

The amount of nicotine recovered after 100, 200 and 300 puffs, as well as the overall percent recovery of nicotine, using the ECIG aerosol generator, for channels 1, 2 and channels 1 and 2 combined are shown in Figure 11. There are no significant differences in the nicotine recovered between channels 1 and 2, but the amount of nicotine recovered between 100, 200 and 300 puffs displayed a significant dose-dependent increase (Figure 11, top panels). The amount of nicotine recovered after 0, 100, 200 and 300 puffs of the ECIG aerosol generator forms a linear relationship with R^2^ values of 0.9465, 0.9462 and 0.9447 for channels 1, 2 and channels 1 and 2 combined, respectively (Figure 11, middle panels). Based on the amount of nicotine aerosolized per puff, the overall nicotine percent recovery was 26.39 and 27.91% for channels 1, 2 and channels 1 and 2 combined, respectively (Figure 11, bottom panels).

## 4. Discussion

Commercial ECIG vaping machines and ECIG aerosol generators are currently available, such as the Borgwaldt LM4E (Borgwaldt-Kc, Hamburg, Germany) [8,24], and the Cerulean SM450e (Milton Keys, UK) [9,25], with other machines slowly entering the market, such as the Electronic Cigarette Aerosol Generator (ECAG) developed at the NYU School of Medicine (Central Valley, NY, USA) [10,26] and sold by CH Technologies (Westwood, NJ). These machines tend to be expensive and unaffordable for most laboratories, consequently leading to the development of ‘in-house’ fabricated devices utilizing equipment and supplies commonly found in the lab [11,12]. These improvised setups generally lack the control and stability required for consistent aerosol production, both within experiments and between experiments.

A cost comparison of our dual-channel aerosol generator with two of the commercially available ones listed above is as follows. The cost to build our generator: without the DC voltage power supply, peristaltic pumps, and tubing; is approximately $301.96 (Supplemental Table S1). The DC variable voltage power supply used in our system cost approximately $350.00. The pumps, which were already in our laboratory’s inventory cost approximately $1500.00 each. The Tygon^®^ tubing used to transport aerosol cost approximately $126.00 for 25 feet. This brings the total cost of our aerosol generator system to $3777.96. In contrast, the five port Borgwaldt LM5E (which has replaced the LM4E) and the one port ECAG sell for approximately $48,500 and $18,200, respectively, as quoted from representatives of the two companies. A quote for the 20-channel Cerulean SM450e was never received.

Consistent production of aerosol is dependent on three factors. First, the ECIG generator must be free of human timing errors, thus the need for programmable automation of puff topography. Secondly, the ECIG generator must maintain a constant airflow (determined by pump flow rate). Finally, the ECIG generator requires a consistent energy supply. Soulet et al. [27] point out that ECIG batteries “can discharge and have limited power delivery”, ultimately impacting aerosol production. To avoid the effects of battery drain, they developed a machine called “Universal System for Analysis of Vaping (U-SAV)”, with the ability to control electrical energy delivery using an internal generator. The U-SAV is quite elaborate. It can generate aerosol from six channels, allowing for measurements of electrical parameters when using both external batteries and an internal energy source, and can control the rate of aerosol airflow. In contrast, while our ECIG generator is more cost effective, it is limited to maintaining constant puff topography and power delivery to two channels. To maintain constant aerosol airflow, the pumps in our system need to be manually calibrated to the required airflow before the start of each experiment. Regardless of these differences, our system delivers relatively consistent aerosol production over 300 consecutive puffs, provided the coil resistors within the atomizers remain in pristine condition.

According to an online review [28], the life of a TFV16 Mesh 0.17 Ω coil is approximately three days depending on the type of E-liquid used and amount of E-liquid in the tank. High vegetable glycerine juices and sweet flavors can significantly shorten the life of the coil. Furthermore, individuals have their own unique vaping/puff topographies which contributes to the complexity of coil lifetime. The short life expectancy of the TFV16 Mesh 0.17 Ω coil [28], as well as variations in the resistance of the coils resulting from the manufacturing process [29,30], could account for the variations in the electrical parameters we noted over time (i.e., 300 consecutive puffs) within a channel and between channels. Regardless, discovering imperfections associated with coil production is not the aim of this study. Albeit that variations within the same brand of resistance coils can alter aerosol production, the aim of this study is to see how the coils connected to our ECIG aerosol generator fare in terms of producing consistent aerosol production over a period of 75 min (300 puffs). Therefore, variations attributed to coil manufacturing must be taken into account as normal variation within an experiment’s design.

### 4.1. Temperature Profiles

High atomizer temperatures can be an issue, particularly when puffing continuously (4 puffs/minute for 75 min or 300 puffs). High temperatures make it uncomfortable to handle the atomizer and, more importantly, are known to generate harmful chemicals which are released with the ECIG-generated aerosol [29,30,31,32,33]. For this reason, the target wattage of the ECIG generator was set at ≈120 W or 60 W for each atomizer in parallel, despite the company’s recommendation of 120 W for each atomizer. In vaping parlance, wattage is directly correlated with heat production [34]. Consequently, a controlled voltage value of 3.811 V was used to achieve the target wattage and hence lower atomizer temperature.

At these settings, inside and outside temperatures of the atomizers after 100 puffs plateaued between 72 and 76 °C and between 54 and 56 °C, respectively (Figure 4 and Table 1). While these temperatures are high, they are a culmination of continuous puffing for 75 min. In contrast, after the first three minutes of puffing (or 12 consecutive puffs) the inside and outside atomizer temperatures increased to ~55 and 35 °C, respectively, which are more realistic temperatures associated with normal sub-ohm (resistance coils < 1 Ω) vaping. At plateau, temperatures varied little within the channel (average difference of <1.0 °C between the second and third sets of 100 puffs). In comparison, plateau temperatures between channel 1 and channel 2 displayed significant variation (average difference of ~4.0 °C between channel 1 and channel 2 after the second and third sets of 100 puffs). It is unclear why this temperature difference exists other than to say the two atomizer coils are inherently different [28], including manufacturing variations that could impact the coil’s resistance [29,30]. Below, a comparison is made between the current study and three other studies [31,32,33] found in the literature. 

Sleiman et al. [31], reported the aerosol temperatures of two ECIG devices, an eGO CE4 (voltage = 3.8 V; coil resistance = 2.6 Ω) and a Kangertank Aerotank Mini (voltage = 3.8 V; coil resistance = 2.0 Ω). Temperatures were monitored using K-type thermocouples placed inside the ECIG device’s mouthpiece making sure to avoid contact with the wall of the device. Using a puffing protocol of 50 puffs over 25 min (5 s puffs, 25 s interpuff duration and 50 mL puff volume) they determined that the eGO and Kangertank devices reached temperatures of 34 and 30 °C, respectively. As voltage on the eGO device was allowed to increase from 3.3 to 4.8 in 0.5 V increments, the temperature of the aerosol also increased in a voltage-dependent manner from approximately 31 to 39 °C with a maximum power output of 8.8 W. Consequently, the temperature of their devices must be lower than what we report for inside the atomizer and more akin the outside atomizer temperature. The reason for this is likely due to thermocouple placement in the ECIG device and that the power output of the eGO and Kangertank devices are both much lower than the TFV16 Smok^®^ tanks used in the current study.

Geiss et al. [32], used an Eleaf Istick Mod and a Kayfun 3.1 atomizer equipped with a 1.6 Ω nichrome coil to record atomizer core temperatures. They monitored temperatures using a FLIR thermal camera at four wattage/voltage settings (5 W/3.0 V, 10 W/4.2 V, 15 W/5.0 V and 20 W/5.6 V). Puff topography consisted of 3 s puffs every 20 s for 2 min (five puffs total) with a puff volume of 50 mL/puff. Atomizer core temperatures increased directly with both power output and voltage. Furthermore, each successive puff yielded slightly higher temperatures. At the end of five puffs, a setting of 20 W/5.6 volts with a coil resistance of 1.6 Ω yielded a core temperature of over 300 °C. This temperature is much higher than what we report in the current investigation. The means by which temperature values were collected is a likely explanation for this difference. With thermal imaging, temperature is more closely determined at the resistance coil itself compared to use of K-type thermocouples which can only be placed as close as feasibly possible to the coils.

Beauval et al. [33] used a Mod box TC (power source set to 18 or 30 W) and an “Air Tank” atomizer equipped with a 0.5 Ω Kanthal coil. They recorded temperatures at the outlet of the ECIG drip tip during twenty 3 s puffs (2 puffs per minute: 55 mL/puff). The temperature profiles they report are like ours, with the first five puffs showing a rise in temperature followed by a plateau. At 18 and 30 W, the temperatures plateaued at 55 and 65 °C, respectively. When puff frequency was increased to 4.3 puffs per minute, the temperatures plateaued at 90 and 115 °C, respectively. In comparison, the temperatures we report in the current study are lower, but Beauval et al. [33], do not report the voltage supplied by the Mod box TC, a factor which could account for this temperature difference. Furthermore, the resistance of the coils they used in their study (0.5 Ω) is more than twice the resistance of coils used in our study (0.17 Ω), which could also contribute to the temperature differences.

### 4.2. Electrical Parameters

A multimeter was used to measure the resistance across two TFV16 Mesh coils connected to the ECIG aerosol generator in parallel, and a discrepancy was noted between the stated resistance on the coil (0.17 Ω) and the actual resistance (0.24 Ω/coil). Since the target power output for the ECIG aerosol generator was 120 W (60 W/channel), and the measured resistance across two coils was 0.12 Ω (0.24 Ω/coil), it was determined that 3.8 V be supplied to the generator. The amperage range for most digital multimeters, including ours, is between 0.001 to 10 A. Consequently, when the voltage is set at 3.8 V, current across any coil with a resistance less than 0.38 Ω, current cannot be directly measured via a standard multimeter. This is the case with the TFV16 Mesh 0.17 Ω coils used in this investigation.

Many of the other techniques for directly measuring the current would add components to the circuit and thus impact the electrical parameters leading to increased uncertainties in the dissipated power by the coils. To circumvent this issue, current was measured indirectly using the well-known relation between the current in a coil and the magnetic field generated by that current. This generated magnetic field was then measured using a Hall-effect sensor, thus allowing for continuous monitoring of current during every puff. This technique is the basis for the standard clamp ammeters used in high current measurements with standard geometries. The unique geometry requirements for this device led to a home-built measurement setup. Interestingly, the same set of measurement considerations is found by groups researching renewable energy production such as solar or wind powered systems [35]. Figure 2 illustrates the high correlation between magnetic field and current (R^2^ values of 0.9598 and 0.9899 for channels 1 and 2, respectively). Given that voltage (V) is constant at 3.8 V, all current values determined with every puff (~60 values per 3 s puff), were used to back calculate resistance and power for up to 300 puffs. In other words, resistance (Ω) and power (W) reflect the current (A) determined from the correlation of magnetic field. For all electrical parameter experiments, the back calculations of resistance (Ω = V/A) ranged from 0.181 to 0.224 Ω across all electrical parameter experiments, and the back calculations of power (W = V^2^/ Ω) ranged from 66 to 80 W. The discrepancies observed between the direct (0.24 Ω) and back calculated resistances could be attributed to background interference in magnetic field created by surrounding electrical devices which were impossible to entirely eliminate in the laboratory environment. Anecdotally, we found, during the initial testing of the current/magnetic field measurements, that the magnetic field generated by the running motor of a small fan in the lab room overlayed a standard 60 Hz oscillation onto the data produced by the Hall-effect sensor. Sources of background magnetic fields such as the fan were removed, when possible, but, given the widespread use of electronics and electrical systems at any given time, some variation in the background field will likely be present.

The electrical parameters of the ECIG aerosol generator in both experiments (the first five puffs experiment and the 300 continuous puffs experiment) remained remarkably stable within each channel (Figure 5 and Figure 6 and Table 2 and Table 3). In channel 1, the current significantly increased only after 200 puffs (from 19.2 to 21.1 A), but in channel 2, the current remained consistent (from 17.1 to 17.2 A) throughout 300 puffs. One explanation for the increase in current after 200 puffs in channel 1 is a puff-related deterioration of the coil resulting in decreased resistance just before the coil fails. Saleh et al. [29] observe this age-related decline in the resistance of the coils used in the Vuse ALTO pods just before the coils fail and they attribute coil failure to the absence of E-liquid in the pod. Vuse ALTO pods are small disposable tanks containing roughly 1.8 mL of E-liquid. Once the liquid is gone, the pods are thrown away. For the Vuse ALTO pods the initial resistance of the coils ranged from 0.89 to 1.14 Ω, and the number of puffs to coil failure was determined to be 158 ± 22. The previously mentioned work by Geiss et al. [32] present power/voltage data that indicate a decrease in the coil resistance as the measurements progressed to higher power/voltages. The coil resistance calculated from the lowest power settings indicate a resistance of approximately 1.8 Ω, while decreasing to slightly below 1.6 Ω at the highest power settings. A calculation of the resistance of the coil based upon the material specifications given in their work (nichrome wire 11 cm in length and 3.0 mm diameter) indicate a nominal value of 1.75 Ω, prior to any degradation from use. This is illustrative of the deterioration of the coils exhibited by decreased resistance.

Manufacturing variations [29,30] of the coils could account for the differences in coil resistance and hence the differences we observed in currents between channels. With a trend towards sub-ohm vaping, even small manufacturing variations could significantly impact coil resistances [30]. Since a total of four different coils were used in the first five puffs experiment (one coil for each channel) and the 300 continuous puffs experiment (one coil for each channel), this would also explain why currents after the first five puffs were significantly different than currents after 100 puffs within each channel. At initial use, a calculation of mean resistance (µ = 0.210 Ω) and its standard deviation (σ = 0.009 Ω) for the four TFV16 Mesh coils yielded a coefficient of variation (CV) of 4.5% and is similar to what was reported for the Vuse Alto pod (µ = 1.031, σ = 0.52, CV = 5.0%, n = 20) by Saleh et al. [30]. Assuming normal distribution, where 99.9% of all resistances fall within 3 σ units of µ, manufacturing imperfections in the TFV16 Mesh coils could account for as much as 13.5% variation in the coil’s resistance. Overall, despite the differences observed between channels, the electrical parameters observed within channels remained remarkably stable, especially within 200 continuous puffs. This suggests that manufacturing variations of the TFV16 Mesh coils are a major contributor to the variations in electrical parameters observed both within and between channels when multiple resistance coils are used. Unfortunately, there is no way around this problem since the life span of each resistance coil is limited and thus needs to be replaced frequently.

### 4.3. Particle Distributions

The average diameter of ECIG-generated aerosol particles ranges from <0.05 to >1.0 µm, with most particles falling between 0.1 and 0.2 µm [15,36,37,38,39]. Furthermore, particle size distribution profiles are known to shift depending on the temperature of the coil, the power (dependent on coil resistance), the wetness of the coil, and the E-liquid composition [15,39,40,41]. The instrumentation used in the current investigation (the PCE-PCO 2 particle counter) is limited in its capacity to measure particle sizes < 0.3 µm. Nonetheless, the results of our study support what has been reported in the literature as indicated by the progressive increase observed in particle counts when particle size decreases from 10 to 0.3 µm. It is highly likely that particle counts for particle sizes < than 0.3 µm (if measured in our system) would be even higher. Interestingly, an increase is also observed in particle counts as particle size increases from 1.0 to 2.5 µm, followed by a decrease as particle size increases from 2.5 to 10 µm. Taken together, the results of this study indicate a likely peak in particle counts at <0.3 µm and a second, but smaller, peak in particle counts between 1.0 and 5.0 µm. This second peak in particle counts has also been observed by Floyd et al. [15], at ≈1.0 µm.

While minute to minute variations in the particle counts for all particle sizes display a great deal of variation (Figure 7), these counts remain relatively stable throughout 300 continuous puffs (Table 4), with significant variation occurring after 100 puffs. For both channels there was a progressive increase in particle counts for all particle sizes. Within Channel 1, particle counts for particle sizes 0.5, 1.0, 2.5 and 5.0 µm significantly increased between the first, second and third sets of one hundred puffs. Within Channel 2, only particle counts for particle size 5.0 µm significantly increased between the first, second and third sets of one hundred puffs. This gradual increase in particle counts over 300 continuous puffs could be a result of decreased coil resistance over time, consequently increasing power and temperature which impacts aerosol production [15,39,40]. After 100 puffs, there was no difference between channel counts for all particle sizes. After 200 puffs, between-channel differences in counts appeared for particle sizes 5.0 and 10 µm. After 300 puffs, between-channel differences in counts appeared for particle sizes 0.5, 1.0, 5.0 and 10 µm. These results indicate that no significant variation, both within and between channels, occurs for up to 100 continuous puffs by the ECIG aerosol generator. Our second experiment (Figure 8 and Table 4), in which particle counts for all six particle sizes were determined during three consecutive sets of 12 puffs in fifteen-minute intervals, supports this claim, as indicated by the lack of significance within and between channels.

Like particle counts, minute to minute variations in the mass concentration data for particle sizes 2.5 and 10 µm also display a great deal of variation (Figure 7), but these concentration data also appear to be relatively stable throughout 300 continuous puffs (Table 4). Significance within channels was only observed for Channel 2, in which the concentration of the 10 µm particle size significantly increased after 300 puffs. Between-channel variations confirm decreased stability after 100 puffs. The second experiment (Figure 9 and Table 4), in which mass concentration for 2.5 and 10 µm particle sizes were determined during three consecutive sets of 12 puffs in fifteen-minute intervals, again supports the claim that this ECIG aerosol generator is consistent in its production of aerosol within 100 puffs. Of note is the observation that particle count for the 2.5 µm particle size is higher than the 10 µm particle size. In contrast, the mass concentration for the 2.5 µm particle size is lower than the 10 µm particle size. This reversal is likely attributed to the hygroscopic nature of the E-liquid, glycerol in particular [42], allowing for particle growth [15]. Particle growth occurs when smaller particle sizes coalesce into larger particle sizes, thus contributing to the increase in mass concentration of the larger particle sizes. Furthermore, particle growth may be a major contributor of the second, but smaller, peak observed at 2.5 µm particle size. As a final note, the observed variations for both particle count and mass concentration profiles (Figure 7) is typical of vaping in an enclosed space, as reported by Borgini et al. [43]. The difference between the current investigation and the Borgini et al. [43] study is that humans were allowed to vape freely (10 puffs over three to six seconds) in a 48 m^3^ room with 0.7/0.8 air changes per hour (as opposed to four machine-generated puffs per minute within a small semi-confined space). Even with these differences between the two studies, particles/cm^3^ peaks observed in the Borgini et al. [43] study show similar variations as compared to the current study. However, their particle counts are lower, and their peaks have a sharper resolution, undoubtedly due to faster aerosol dissipation because of a larger enclosed space.

### 4.4. E-Liquid Vaporization and Aerosol Delivery

Using the weight differences of the atomizer plus the E-liquid (before and after puffing on the ECIG aerosol generator) and the density of the E-liquid (1.18 g/mL), the volumes of aerosolized E-liquid were determined after 100, 200 and 300 puffs for both channels (Table 5). Linear regression analysis (Figure 10), correlating puff number with volume of E-liquid aerosolized, revealed a linear relationship with R^2^ values of nearly one for both channels (with and without forcing the line through the origin). These results indicate a high level of consistency and repeatability within and between channels. From the linear equations, the amount of E-liquid vaporized was determined to be 19.00 and 19.33 µL/puff for channels 1 and 2, respectively, with an average of 19.17 µL/puff (~22.6 mg E-liquid consumed per puff). Consequently, the small, but significant variations observed (between channels) in the atomizer temperatures and in the electrical parameters did not significantly affect the amount of aerosol delivered (as indexed by nicotine concentrations) for up to 300 puffs. Ultimately, it is the amount of aerosol delivered within an experimental protocol that will yield biochemical and physiological consequences. The amount of E-liquid vaporized is highly dependent on the E-liquid composition and puff topography [44,45], but it is also highly influenced by the supplied power and, hence, temperature. The amount of E-liquid vaporized is directly proportional to supplied power [15,45,46]. Soulet et al. [46] tested several atomizers ranging from 0.5 to 1.8 Ω, with power settings ranging from 11 to 50 W. The Cubis 1 Ω atomizer (set at 15 W) consumed 9.29 mg per 3 s puff. This is comparable to 9.3 µL/puff (~11.0 mg E-liquid consumed per 5 s puff) for the Triple3 eGO device (2.6 Ω, 3.7 V and ~5.7 W) determined in a previous study from our laboratory [12]. When Soulet et al. [46] analyzed four sub-ohm atomizers (each at 0.5 Ω), the amount of E-liquid consumed increased proportionately to more than 17 and 23 mg/puff, for the CL and MII tanks, respectively, when the power supply was increased to 50 W. Again, these E-liquid consumption values are comparable to what was determined for our ECIG aerosol generator using the TFV16 SMOK^®^ atomizer with a stated 0.17 Ω coil and a power setting of ~60 W.

In this study, nicotine (20 mg/mL E-liquid) in aerosol is used as a dosimetry marker to assess aerosol delivery after 0, 100, 200 and 300 puffs on the ECIG aerosol generator [47]. The assumption is made that the amount of E-liquid vaporized by the atomizers is proportional to the amount of nicotine delivered to the BHI growth media via the aerosol. Despite significant variations in aerosol particle distributions, both within and between channels, aerosol delivery remains remarkably stable. It is important to point out here, that aerosol particle distribution and aerosol delivery are not the same thing. Aerosol particle distribution is constantly changing depending on the dynamics within the semi-confined space where particle counts/concentrations were measured. Particle counts/concentrations were determined after monitoring for one minute, for a total of 75 consecutive minutes (or 300 puffs). During each minute, a total of four puffs is delivered to the semi-confined space, with the particles of one puff interacting (i.e., colliding and coalescing) with particles of a subsequent puff. What this means is that the particle distribution for each size is ever changing (hence the variability), while the overall amount of total aerosol delivered remains more stable. The nicotine delivered from the atomizers is consistent as evidenced by the high linear correlation (R^2^ = 0.9464 and 0.9462 for Channels 1 and 2, respectively, with combined R^2^ = 0.9447) of the amount of nicotine recovered from the BHI over the 300-puff period (Figure 11 top and middle panels). Furthermore, no statistical differences are noted between channels. When nicotine recovery is normalized to the amount of E-liquid vaporized on a per puff basis (Figure 11 bottom panels), the average percent recovery of nicotine is >25% (range from 26.4 to 27.9 %). The primary reasons for nicotine loss include (1) E-liquid lost as undissolved aerosol vented from the 50 mL conical tube and (2) condensation of E-liquid in the outlet tubing extending from the atomizers to the 50 mL conical tubes (Figure 1F). In a previous study [22], using manually operated peristaltic pumps and ECIG devices, the nicotine percent recovery was lower, ranging from 8.4 to 10.1 %. One reason the current study has a higher percent recovery is the surface area to volume ratio of the outlet tube is ~4.5 times less, thus allowing for less condensation of E-liquid on the inner wall of the tubing [21,22,23]. A second factor contributing to higher percent recovery in the present study includes the increased flow rate from 0.4 mL/minute (33 mL per 5 s puff) to 1.1 mL/minute (55 mL per 3 s puff), thus allowing less time for condensation of E-liquid to occur [21,22,23]. Another contributing factor to the higher percent recovery could be the higher aerosol temperature generated by using a sub-ohm tank.

Several studies, as reviewed by Smart and Phillips, (2020) [48], have used the bubble-through method for trapping of nicotine and other substances present in ECIG-generated aerosol in a variety of aqueous culture media. With that said, it is difficult to make direct comparisons of nicotine percent recoveries due to the myriad of methodological variations. While direct comparisons cannot be made, it is evident that nicotine recovery yields vary using bubble-through aqueous extraction techniques. Table 6 attempts to make comparisons between this study and others using the bubble-through technique. For example, Taylor et al. [47], Taylor et al. [49] and Breheny et al. [50] found nicotine yields between 4 and 5 µg/mL after bubbling ten puffs through 20 mL of Dulbecco’s Modified Eagle’s Medium (DMEM) or VascuLife capture media. All three studies used a Vype ePen ECIG device with an initial nicotine concentration of 18 mg/mL or an unspecified closed modular device. Using the Health Canada Smoking Regimen [51] of 55 mL per 2 s puff every 30 s, Munakata et al. [52] report a nicotine return of 314.4 µg/mL following 300 puffs in 15 mL of Dulbecco’s Modified Eagle’s Medium. Unfortunately, neither the ECIG device used nor the initial concentration of nicotine in the E-liquid was ever specified. In comparison, our study finds 81 or 1002 ug/mL of nicotine (as determined from linear regression analysis; Figure 11, middle panel, combined) after bubbling 10 or 300 puffs into 10 mL of BHI media, respectively. The higher nicotine yields reported in the current study are not unexpected since sub-ohm puffing is known to produce significantly higher aerosol nicotine yields when coil resistance decreases and power supply increases. Noel et al. [53] found a 7.2-fold increase in levels of aerosolized nicotine as the resistance of the atomizer coil decreased. At 4.8 V, nicotine levels were approximately 150, 400 and 1000 µg/puff for resistances of 1.5, 0.5 and 0.15 Ω, respectively. Floyd et al. [54] reported nicotine yields of 66.6 165.6 and 198.4 µg/puff at 25, 50 and 75 W, respectively, using an atomizer with 0.5 Ω coil resistance.

## 5. Conclusions

The ECIG aerosol generator, built and tested at Lincoln Memorial University and described within this manuscript, can provide consistent and reliable aerosol for analysis in numerous experimental procedures. Puff number, puff duration, and puff interval are programmable, thus eliminating the human interphase for each trial run. Furthermore, the device is an economical alternative to commercially available aerosol generators on the market. However, this machine is not without its limitations. As described, this aerosol generator can run one channel (i.e., one atomizer) or two channels (i.e., two atomizers simultaneously) in parallel. Therefore, if dual channels are operated simultaneously, each channel is limited to operating under the same voltage, current and power conditions. Any atomizer with a 510 connector can be used with the device, and the coils within these atomizers complete the electrical circuitry of the aerosol generator. While the atomizers are not part of the ECIG aerosol generator per se, the coils within them must be carefully monitored since manufacturer variations, puff topography and the absence or presence of E-liquid can affect the coil’s longevity and performance, ultimately affecting aerosol production. As an additional precaution, the ECIG aerosol generator should be shielded (as much as possible) from nearby electrical equipment since they are known to create magnetic fields (Hall-effect sensor), and, hence, extraneous currents, which can interfere with the machine’s performance, particularly the characterization of its performance.

Despite these drawbacks, the ECIG aerosol generator described within this paper produces consistent and reliable aerosol on a per puff basis for at least 100 puffs (four-55 mL puffs per minute) as verified by the stability in (1) atomizer/coil temperatures, (2) electrical parameters of the device itself, (3) distribution of aerosol particles within a confined space, (4) E-liquid vaporization volume and (5) aerosol delivery (as indexed by nicotine recovery). Between-channel variations are greater than within-channel variations for all criteria measured, a possible consequence of the inherent differences associated with each resistance coil. At greater than 100 puffs, within- and between-channel variations are magnified. It should be noted that all criteria determined in this investigation were done so at twice the puff number recommended by CORESTA guidelines [7]. If CORESTA guidelines (two-55 mL puffs per minute) were followed, it is likely that the ECIG aerosol generator would have produced consistent and reliable aerosol for more than 100 puffs.

## Figures and Tables

**Table 1:** Temperature Profiles.

Temperatures	Channel 1			Channel 2			Channel 1 vs. Channel 2 (*p* Values)		
	0 to 100 Puffs (n = 300)	100 to 200 Puffs (n = 299)	200 to 300 Puffs (n = 299)	0 to 100 Puffs (n = 300)	100 to 200 Puffs (n = 299)	200 to 300 Puffs (n = 299)	0 tor 100 Puffs	100 to 200 Puffs	200 to 300 Puffs
Inside Temp. (°C)	68.3 ± 0.6 *^,@,#^	76.2 ± 0.2	75.4 ± 0.2	62.7 ± 0.6 ^@,#^	72.0 ± 0.1	71.6 ± 0.1	*p* < 0.001	*p* < 0.001	*p* < 0.001
Outside Temp. (°C)	45.9 ± 0.5 ^@,#^	56.0 ± 0.1	56.6 ± 0.0	47.6 ± 0.4 ^@,#^	54.1 ± 0.0	54.1 ± 0.0	*p* < 0.001	*p* < 0.001	*p* < 0.001

* = mean ± SEM of all temperature values over the designated puff interval; ^@^ = *p* < 0.05 as compared to 200 puffs within the same channel; ^#^ = *p* < 0.05 as compared to 300 puffs within the same channel.

**Table 2:** Statistics of Magnetic and Electrical Coil Properties after the First Five Puffs.

	Magnetic Field (mT)		Currents (A) ^@^		Resistance (Ω) ^#^			Power (W) ^^^		
	Channel 1	Channel 2	Channel 1	Channel 2	Channel 1	Channel 2	Stated on Coil ^!^	Channel 1	Channel 2	Stated on Coil
Mean (n = 503)	0.0316 *	0.0305	18.4 *	18.0	0.208 *	0.212	0.17 Ω	70.1 *	68.6	Best at
Standard Deviation	0.0012	0.0013	0.7	0.7	0.008	0.009		2.6	2.8	120 W
Standard Error	0.0001	0.0001	0.0	0.0	0.000	0.000	Direct Measure ^$^	0.1	0.1	Rated between 80 to 160 W
							0.24 Ω			

^@^ = correlation from magnetic field; ^#^ = calculated from current and a voltage of 3.8 V; ^^^ = calculated from resistance and voltage of 3.8 V; ^!^ = stated on the Smok^®^ TFV16 Mesh Coil and packaging; ^$^ = direct measurement using a multimeter; * = *p* < 0.001 between channel 1 and channel 2.

**Table 3:** Electrical Parameters.

**Electrical** **Parameters (A)**	**Channel 1**			**Channel 2**			**Channel 1 vs. Channel 2 (*p* Values)**		
	**0 to 100 Puffs (n = 303)**	**100 to 200 Puffs (n = 305)**	**200 to 300 Puffs (n = 304)**	**0 to 100 Puffs (n = 303)**	**100 to 200 Puffs (n = 305)**	**200 to 300 Puffs (n = 304)**	**0 tor 100 Puffs**	**100 to 200 Puffs**	**200 to 300 Puffs**
Magnetic Field (mT)	0.0331 ± 0.0001 *^,#^	0.0333 ± 0.0001 ^#^	0.0362 ± 0.0001	0.0292 ± 0.0001	0.0289 ± 0.0001	0.0290 ± 0.0001	*p* < 0.001	*p* < 0.001	*p* < 0.001
Current (A)	19.2 ± 0.0 ^#^	19.4 ± 0.0 ^#^	21.1 ± 0.0	17.2 ± 0.0	17.1 ± 0.0	17.2 ± 0.0	*p* < 0.001	*p* < 0.001	*p* < 0.001
Resistance (Ω)	0.198 ± 0.000 ^#^	0.197 ± 0.000 ^#^	0.181 ± 0.000	0.221 ± 0.001	0.224 ± 0.001	0.221 ± 0.001	*p* < 0.001	*p* < 0.001	*p* < 0.001
Power (W)	73.3 ± 0.2 ^#^	73.9 ± 0.2 ^#^	80.3 ± 0.2	65.7 ± 0.2	65.0 ± 0.2	65.4 ± 0.2	*p* < 0.001	*p* < 0.001	*p* < 0.001
**Electrical** **Parameters (B)**	**Channel 1**			**Channel 2**					
	**First 5 Puffs from Table 2 (n = 503)**	**After 100 Puffs from** **above (n = 303)**		**First 5 Puffs (n = 503)**	**After 100 Puffs from** **above (n = 303)**		**First 5 Puffs**	**After 100 Puffs**	
Magnetic Field (mT)	0.0316 ± 0.0001 ^^^	0.0331 ± 0.0001		0.0305 ± 0.0001 ^^^	0.0292 ± 0.0001		*p* < 0.001	*p* < 0.001	
Current (A)	18.4 ± 0.0 ^^^	19.2 ± 0.0		18.0 ± 0.0 ^^^	17.2 ± 0.0		*p* < 0.001	*p* < 0.001	
Resistance (Ω)	0.208 ± 0.000 ^^^	0.198 ± 0.000		0.212 ± 0.000 ^^^	0.221 ± 0.001		*p* < 0.001	*p* < 0.001	
Power (W)	70.1 ± 0.1^^^	73.3 ± 0.2		68.6 ± 0.1 ^^^	65.7 ± 0.2		*p* < 0.001	*p* < 0.001	

A = electrical parameters during after the first, second and third sets of 100 puffs; B = comparison of electrical parameters after the first five puffs with the electrical parameters after 100 puffs. * = mean ± SEM of all electrical parameter values over the designated puff interval; ^#^ = *p* < 0.05 as compared to 300 puffs within the same channel; ^^^ = *p* < 0.05 as compared to 100 puffs for the same channel.

**Table 4:** Particle Counts and Mass Concentrations.

**Experiment 1** **300 Consecutive Puffs**	**Channel 1**			**Channel 2**			**Channel 1 vs. Channel 2 (*p* Values)**		
	**0 to 100 Puffs (n = 26)**	**100 to 200 Puffs (n = 25)**	**200 to 300 Puffs (n = 25)**	**0 to 100 Puffs (n = 26)**	**100 to 200 Puffs (n = 25)**	**200 to 300 Puffs (n = 25)**	**0 to 100** **Puffs**	**100 to 200 Puffs**	**200 to 300 Puffs**
**Particle Count**									
0.3 µm (particles/ft ^3^)	264,415 ± 18,162 *	245,322 ± 16,755	284,808 ± 10,319	258,144 ± 7063	286,950 ± 10,115	256,517 ± 7872	NS	NS	NS
0.5 µm (particles/ft ^3^)	206,922 ± 13,836 ^@,#^	248,815 ± 6038 ^#^	293,491 ± 9031	206,613 ± 13,860	228,549 ± 5446	235,091 ± 6789	NS	NS	*p* < 0.001
1.0 µm (particles/ft ^3^)	43,202 ± 4054 ^#^	56,159 ± 2585 ^#^	70,512 ± 3150	44,561 ± 3859	49,352 ± 2562	51,448 ± 3428	NS	NS	*p* < 0.01
2.5 µm (particles/ft ^3^)	77,581 ± 6082 ^#^	92,067 ± 3838	98,047 ± 2607	89,687 ± 7612	107,573 ± 2440	108,615 ± 2694	NS	NS	NS
5.0 µm (particles/ft ^3^)	679.2 ± 70 ^@,#^	1317 ± 151 ^#^	2046 ± 89	724 ± 73 ^@,#^	818 ± 52^#^	859 ± 56	NS	*p* < 0.01	*p* < 0.001
10.0 µm (particles/ft ^3^)	1209 ± 104	1271 ± 54	1369 ± 60	1199 ± 97	1955 ± 148	2622 ± 89	NS	*p* < 0.001	*p* < 0.001
**Mass Concentration**									
2.5 µm (µg/m^3^)	1526 ± 180	1018 ± 64	1372 ± 80	1531 ± 133	2029 ± 190	2746 ± 159	NS	*p* < 0.001	*p* < 0.001
10.0 µm (µg/m^3^)	5249 ± 606	3885 ± 331	5922 ± 504	5281 ± 596 ^#^	6521 ± 570	8446 ± 386	NS	*p* < 0.01	*p* < 0.01
**Experiment 2** **3 sets of 12 puffs**	**Channel 1**			**Channel 2**			**Channel 1 vs. Channel 2 (*p* values)**		
	**First 12 Puffs (n = 15)**	**Second 12 Puffs (n = 15)**	**Third 12 Puffs (n = 15)**	**First 12 Puffs (n = 15)**	**Second 12 Puffs (n = 15)**	**Third 12 Puffs (n = 15)**	**First** **12 Puffs**	**Second** **12 Puffs**	**Third** **12 Puffs**
**Particle Count**									
0.3 µm (particles/ft ^3^)	163,494 ± 29,738	157,120 ± 27,515	155,424 ± 26,053	90,806 ± 25,485	87,848 ± 21,593	78,751 ± 20,548	NS	NS	NS
0.5 µm (particles/ft ^3^)	117,984 ± 23,286	114,452 ± 21,869	113,120 ± 21,082	63,994 ± 18,744	60,827 ± 16,020	56,611 ± 15,654	NS	NS	NS
1.0 µm (particles/ft ^3^)	28,946 ± 5632	27,612 ± 5323	28,154 ± 5349	16,183 ± 4729	15,570 ± 4186	15,009 ± 4216	NS	NS	NS
2.5 µm (particles/ft ^3^)	23,791 ± 6676	26,958 ± 7659	23,195 ± 6603	13,130 ± 4878	11,866 ± 3959	10,904 ± 3563	NS	NS	NS
5.0 µm (particles/ft ^3^)	690 ± 163	731 ± 182	723 ± 183	521 ± 185	526 ± 178	548 ± 187	NS	NS	NS
10.0 µm (particles/ft ^3^)	1167 ± 245	1276 ± 280	1315 ± 288	797 ± 256	860 ± 270	867 ± 277	NS	NS	NS
**Mass Concentration**									
2.5 µm (µg/m^3^)	307 ± 90	305 ± 79	439 ± 155	527 ± 165	767 ± 298	708 ± 253	NS	NS	NS
10.0 µm (µg/m^3^)	845 ± 247	1023 ± 294	1328 ± 439	1425 ± 427	1897 ± 596	1724 ± 551	NS	NS	NS

* = mean ± SEM of all particle count and mass concentration values over the designated puff interval; ^@^ = *p* < 0.05 as compared 200 puffs within the same channel; ^#^ = *p* < 0.05 as compared 300 puffs within the same channel; NS = not significant.

**Table 5:** Volume of E-liquid Aerosolized per Puff of the ECIG Aerosol Generator.

	Channel 1	Channel 2	Combined Channels
Weight of 1 mL E-liquid (g/mL)	* 1.18 ± 0.01 (n = 3)	1.18 ± 0.01 (n = 3)	1.18 ± 0.01 (n = 6)
Tank weight difference (g) before and after 100 puffs	2.26 ± 0.00 (n = 3)	2.18 ± 0.18 (n = 3)	2.22 ± 0.08 (n = 6)
Tank weight difference (g) before and after 200 puffs	4.51 ± 0.12 (n = 3)	4.76 ± 0.06 (n = 3)	4.63 ± 0.08 (n = 6)
Tank weight difference (g) before and after 300 puffs	6.85 ± 0.14 (n = 3)	7.15 ± 0.05 (n = 3)	7.00 ± 0.09 (n = 6)
Volume (mL) aerosolized after 100 puffs	1.92 ± 0.00 (n = 3)	1.84 ± 0.15 (n = 3)	1.88 ± 0.07 (n = 6)
Volume (mL) aerosolized after 200 puffs	3.82 ± 0.10 (n = 3)	4.03 ± 0.05 (n = 3)	3.93 ± 0.07 (n = 6)
Volume (mL) aerosolized after 300 puffs	5.80 ± 0.12 (n = 3)	6.06 ± 0.04 (n = 3)	5.93 ± 0.8 (n = 6)
Volume (µL) aerosolized per puff	19.00 ± 0.00 (n = 3)	19.33 ± 0.67 (n = 3)	19.17 ± 0.31 (n = 6)

* Mean ± SEM.

**Table 6:** Comparison of Nicotine Recovery in Culture Media Using Bubble-through Technique.

Study	Culture Media	Puff Topography	Culture Media Volume (mL) for Aerosol Trapping	E-liquid Nicotine Concentration (mg/mL)	Number	Theoretical Maximum Nicotine Concentration (mg/mL) ^@, +^	Nicotine Recovered in Media (mg/mL) ^^^	Percent Recovery (%)
	Brain Heart Infusion	CORESTA [7] Sub-Ohm ^#^ Atomizer Tank (open)	10	20	10 100 200 300	0.378 3.426 5.543 7.302	0.081 0.673 1.331 1.989	22 20 24 27
Taylor et al. (2016) [47]	Dulbecco’s Modified Eagle Medium	CORESTA [7] Vype ePen (Cigilike) Modular Devise (Closed Tank)	20 20	36 18	10 10	0.342 0.171	0.005 0.005	1 3
Taylor et al. (2017) [49]	VascuLife^TM^	CORESTA [7] Vype ePen (Cigilike) Modular Devise (Closed Tank)	20 20	36 18	10 10	0.342 0.171	0.004 0.005	1 3
Breheny et al. (2017) [50]	Dulbecco’s Modified Eagle Medium	CORESTA [7] Vype ePen (Cigilike)	20	18	10	0.171	0.005	3
Munakata et al. (2018) [52]	Dulbecco’s Modified Eagle Medium	Health Canada Smoking Regimen [51] (55 mL per 2 s puff every 30 s) Unspecified Cigilike devise	15	Unknown Assume between 18 and 36 mg/mL	300	2.823–5.65	0.31	5 to 11

**^@^** = calculated using the amount of E-liquid vaporized per puff, puff number, initial nicotine concentration and volume of E-liquid and maximal final volume of culture media. **^+^** = assume between 9.3 µL/puff vaporized (for greater than 1 Ω resistance) and 19.2 µL/puff vaporized (for less than 1 Ω resistance). ^^^ = determined from linear regression analysis for the current study or as reported from the literature. **^#^** = the coil in the atomizer used in the current investigation is sub-ohm, all other investigations listed in this table use ECIG devises with coil resistances >1 Ω.

## Data Availability

Not Applicable.

## Supplementary Materials

The following are available online at https://www.mdpi.com/article/10.3390/ijerph182413190/s1, Figure S1: Arduino, Figure S2: Printed Cicuit Board, Figure S3: Schematic of Printed Circuit Board, Figure S4: Computer Application Screen, File S1: Aerosol Generator Computer Code, Table S1: Components of the Aerosol Generator.
