# Supplementary material for: Fabrication and Validation of an Economical, Programmable, Dual-Channel, Electronic Cigarette Aerosol Generator
Source: Int J Environ Res Public Health. 2021 Dec 14;18(24):13190. doi: 10.3390/ijerph182413190 (PMC8703563; doi:10.3390/ijerph182413190)
Supplement: Supplementary file 1 [file ijerph-18-13190-s001.zip › Suppl File S1.pdf]

```
#include <Wire.h>
#include <LiquidCrystal_I2C.h>


LiquidCrystal_I2C lcd(0x3F, 16, 2);
int puffs = 100;   //CHANGE THIS TO CHANGE THE AMOUNT OF PUFFS IN THE CYCLE
int relay = 7;
int pumpRelay = 8;
int fxAbort = 13;
int fxAbort_remote = 0;
int pumpBypass = 12;
int vapeBypass = 11;
int dualBypass = 10;
int fxStart = 9;
int fxStart_remote = 0;
int count = 0;
int statepin10 = 0; // 0 = LOW and 1 = HIGH
int puffInterval = 5000;   //CHANGE THIS TO CHANGE THE TIME BETWEEN PUFFS (MUST BE WRITTEN IN MS)
CANNOT GO BELOW 4100 BECAUSE OF PUMP BOOT TIME
int puffDuration = 5000;   //CHANGE THIS TO CHANGE THE PUFF LENGTH (MUST BE WRITTEN IN MS)
int pumpBoot = 4100;

//NOTE: Many instances of redundancy

String parse_input(String data, char separator, int index)
{
  int found = 0;
  int strIndex[] = { 0, -1 };
  int maxIndex = data.length() - 1;

  for (int i = 0; i <= maxIndex && found <= index; i++) {
    if (data.charAt(i) == separator || i == maxIndex) {
      found++;
      strIndex[0] = strIndex[1] + 1;
      strIndex[1] = (i == maxIndex) ? i+1 : i;
    }
  }
  return found > index ? data.substring(strIndex[0], strIndex[1]) : "";
}


void setup() {
  lcd.begin();
  Serial.begin(9600);
  Serial.print("running");
  pinMode(relay,OUTPUT);
  pinMode(pumpRelay,OUTPUT);
  pinMode(fxAbort,INPUT);
  pinMode(pumpBypass,INPUT);
  pinMode(vapeBypass,INPUT);
  pinMode(dualBypass,INPUT);
  pinMode(fxStart,INPUT);
}
```

```
void loop() {

   if (Serial.available() > 0) {
      String incoming = Serial.readString();
      String incoming_variable = parse_input(incoming, ':', 0);
      int incoming_value = parse_input(incoming, ':', 1).toInt();

      Serial.println(incoming_variable);
      Serial.println(incoming_value);

      if (incoming_variable == "TEST") {
         Serial.println("CHANGING TEST VARIABLE");
      } else if (incoming_variable == "puffcount" ) {
         puffs = incoming_value;
         Serial.println("CHANGING puffcount VARIABLE");
      } else if (incoming_variable == "puffinterval") {
         puffInterval = incoming_value;
         Serial.println("CHANGING puffinterval VARIABLE");
      } else if (incoming_variable == "puffduration") {
         puffDuration = incoming_value;
         Serial.println("CHANGING puffduration VARIABLE");
      } else if (incoming_variable == "pumptimer") {
         pumpBoot = incoming_value;
         Serial.println("CHANGING pumptimer VARIABLE");
      }  else if (incoming_variable == "fxStart") {
         fxStart_remote = 1;
         fxAbort_remote = 0;
         Serial.println("CHANGING fxStart VARIABLE");
      }  else if (incoming_variable == "fxAbort") {
         fxStart_remote = 0;
         fxAbort_remote = 1;
         Serial.println("CHANGING fxAbort VARIABLE");
      } else {
         Serial.println(incoming_variable);
         Serial.println("invalid");
      }
      Serial.flush();
   }


  // put your main code here, to run repeatedly:
  digitalWrite(relay, LOW);
  digitalWrite(pumpRelay, LOW);
  lcd.clear();
  lcd.setCursor(0,0);
  lcd.print("LAST CYCLE: ");
  lcd.print(count);
  lcd.setCursor(0,1);
  lcd.print("PRESS START");
  lcd.setCursor(0,0);
  statepin10 = 0;
  while(digitalRead(dualBypass) == HIGH){
```

```
    Serial.print("Dual Working");
    lcd.clear();
    lcd.print("BYPASSING...");
    lcd.setCursor(0,1);
    lcd.print("KEEP BUTTON HELD");
    lcd.setCursor(0,0);
    if(statepin10 == 0){
      digitalWrite(pumpRelay, HIGH);
      delay(4100);
      digitalWrite(relay, HIGH);
      statepin10 = 1;
      }
   }
  statepin10 = 0;
  digitalWrite(relay, LOW);
  digitalWrite(pumpRelay, LOW);
  while(digitalRead(pumpBypass) == HIGH){
    Serial.print("Pump Working");
    lcd.clear();
    lcd.print("BYPASS: PUMP ON");
    lcd.setCursor(0,1);
    lcd.print("KEEP BUTTON HELD");
    lcd.setCursor(0,0);
    digitalWrite(pumpRelay, HIGH);
    }
  statepin10 = 0;
  digitalWrite(relay, LOW);
  digitalWrite(pumpRelay, LOW);
  while(digitalRead(vapeBypass) == HIGH){
    Serial.print("Tank Working");
    lcd.clear();
    lcd.print("BYPASS: TANK ON");
    lcd.setCursor(0,1);
    lcd.print("KEEP BUTTON HELD");
    lcd.setCursor(0,0);
    digitalWrite(relay, HIGH);
    }
  statepin10 = 0;
  digitalWrite(relay, LOW);
  digitalWrite(pumpRelay, LOW);
  if(digitalRead(fxStart) == HIGH || fxStart_remote == 1){
      Serial.print("Start Working");
      count = 0;
      while(count < puffs){
        lcd.clear();
        lcd.print("PUFFS: ");
        lcd.print(count);
        lcd.setCursor(0,1);
        lcd.print("HOLD TO ABORT");
        lcd.setCursor(0,0);
        digitalWrite(pumpRelay, HIGH);
        delay(pumpBoot);  //PUMP BOOT TIME
        digitalWrite(relay, HIGH);
```

```
        delay(puffDuration);
        count = count + 1;
        lcd.clear();
        lcd.print("PUFFS: ");
        lcd.print(count);
        lcd.setCursor(0,1);
        lcd.print("HOLD TO ABORT");
        lcd.setCursor(0,0);
        if(digitalRead(fxAbort) == HIGH || fxAbort_remote == 1){
          Serial.print("Abort working");
          digitalWrite(relay, LOW);
          digitalWrite(pumpRelay, LOW);
          fxStart_remote = 0;
          fxAbort_remote = 0;
          return;}
        digitalWrite(relay, LOW);
        digitalWrite(pumpRelay, LOW);
        if(count == puffs){
            Serial.print("finished working");
            Serial.print(puffs);
          digitalWrite(relay, LOW);
          digitalWrite(pumpRelay, LOW);
          fxStart_remote = 0;
          fxAbort_remote = 0;
          return;}
        digitalWrite(relay, LOW);
        digitalWrite(pumpRelay, LOW);
        delay(puffInterval - pumpBoot);
          }
          fxStart_remote = 0;
          fxAbort_remote = 0;
        }
}
```
