# Supplementary material for: Fabrication and Validation of an Economical, Programmable, Dual-Channel, Electronic Cigarette Aerosol Generator
Source: Int J Environ Res Public Health. 2021 Dec 14;18(24):13190. doi: 10.3390/ijerph182413190 (PMC8703563; doi:10.3390/ijerph182413190)
Supplement: Supplementary file 1 [file ijerph-18-13190-s001.zip › Suppl Table S1.pdf]

Supplemental Table S1: Components used in the fabrication of a programable dual channel ECIG aerosol generator (not including peristaltic pumps and DC Voltage power supply).

Component	Manufacturer/Brand	Purchased from	Cost
**Arduino UNO Rev. 3**	Arduino	https://www.amazon.com/Arduino-A000066-ARDUINO-UNO-R3/dp/B008GRTSV6/ref=sr_1_3?crid=3TD0I9PSRVU65&dchild=1&keywords=arduino+uno+r3&qid=1627410568&sprefix=arduino%2Caps%2C167&sr=8-3	**$19.99**
**Arduino power supply (9v)**	Corporate Computer	https://www.amazon.com/gp/product/B018OLREG4/ref=ppx_yo_dt_b_asin_title_o08_s00?ie=UTF8&psc=1	**$7.29**
**USB 2.0 A-male to B-male cord (for code upload)**	Amazon Basics	https://www.amazon.com/gp/product/B00BCWALHM/ref=ppx_yo_dt_b_asin_title_o03_s00?ie=UTF8&psc=1	**$6.49**
**I2C LCD display with interface for Arduino**	JANSANE	https://www.amazon.com/gp/product/B07D83DY17/ref=ppx_yo_dt_b_asin_title_o01_s02?ie=UTF8&psc=1	**$9.99**
**120v inlet module (main power)**	Generic Brand (purchased locally from State Electric-Middlesborro, KY)	https://www.stateelectric.com/?gclid=EAIaIQobChMIyeKWqZSG8gIVa_DjBx0D5QK6EAAYASAAEgLnUfD_BwE	**$14.49**
**120v outlets (for pump connection)**	Generic Brand (purchased locally from State Electric-Middlesborro, KY)	https://www.stateelectric.com/?gclid=EAIaIQobChMIyeKWqZSG8gIVa_DjBx0D5QK6EAAYASAAEgLnUfD_BwE	**$3.03**
**2 terminals (two bolts for variable DC power supply)**	Generic Brand (purchased locally from State Electric-Middlesborro, KY)	https://www.stateelectric.com/?gclid=EAIaIQobChMIyeKWqZSG8gIVa_DjBx0D5QK6EAAYASAAEgLnUfD_BwE	**$0.72**
**rocker switch (n=1)**	Jianfeng Store	https://www.amazon.com/QTEATAK-Rocker-Switch-Position-Terminals/dp/B08B84H8WL/ref=sr_1_3?dchild=1&keywords=on%2Foff+rocker+switch+small&qid=1627486367&sr=8-3	**$4.99**
**ky-019 relays (n=2)**	TOKALO	https://www.amazon.com/gp/product/B00VRUAHLE/ref=ppx_yo_dt_b_asin_title_o01_s00?ie=UTF8&psc=1	**$8.80**
**push buttons (n=5)**	EG STARTS	https://www.amazon.com/gp/product/B01MR0E1T6/ref=ppx_yo_dt_b_asin_title_o01_s01?ie=UTF8&psc=1	**$11.99**
**2.2kΩ resistors (n=5)**	AideTek	https://www.amazon.com/gp/product/B007P1DLR6/ref=ppx_yo_dt_b_search_asin_title?ie=UTF8&psc=1	**$0.75**
**510 connectors (for atomizer tanks) (n=2)**	Motley Mods	https://www.motleymods.com/collections/510-connectors/products/pre-soldered-510-connector?variant=13824968032311	**$7.99**
**D1D100K high current solid-state relay**	Sensata/Crydom	https://www.mouser.com/ProductDetail/sensata/d1d100k/?qs=UkDUCjYnTB2s%252bsG%252bXylbQw%3D%3D&countrycode=US&currencycode=USD	**$164.44**
**printed circuit board**	Printed by JLCPCB	https://jlcpcb.com/VGB?gclid=EAIaIQobChMIq9GQ_fGD8gIVVObjBx3yXQnDEAAYASAAEgLSe_D_BwE	**$2.00**

**14 AWG wire**	Generic Brand (purchased locally from State Electric-Middlesborro, KY)	https://www.stateelectric.com/?gclid=EAIaIQobChMIyeKWqZSG8gIVa_DjBx0D5QK6EAAYASAAEgLnUfD_BwE	**$10.00**
**22 AWG wire (various colors)**	EX ELECTRONIX	https://www.amazon.com/gp/product/B00B4ZRPEY/ref=ppx_yo_dt_b_search_asin_title?ie=UTF8&psc=1	**$4.00**
**12” x 12” x 6” plastic junction box**	Generic Brand (purchased locally from State Electric-Middlesborro, KY)	https://www.stateelectric.com/?gclid=EAIaIQobChMIyeKWqZSG8gIVa_DjBx0D5QK6EAAYASAAEgLnUfD_BwE	**$25.00**
			**$301.96**
