# Supplementary figures and images for: Fabrication and Validation of an Economical, Programmable, Dual-Channel, Electronic Cigarette Aerosol Generator
Source: Int J Environ Res Public Health. 2021 Dec 14;18(24):13190. doi: 10.3390/ijerph182413190 (PMC8703563; doi:10.3390/ijerph182413190)

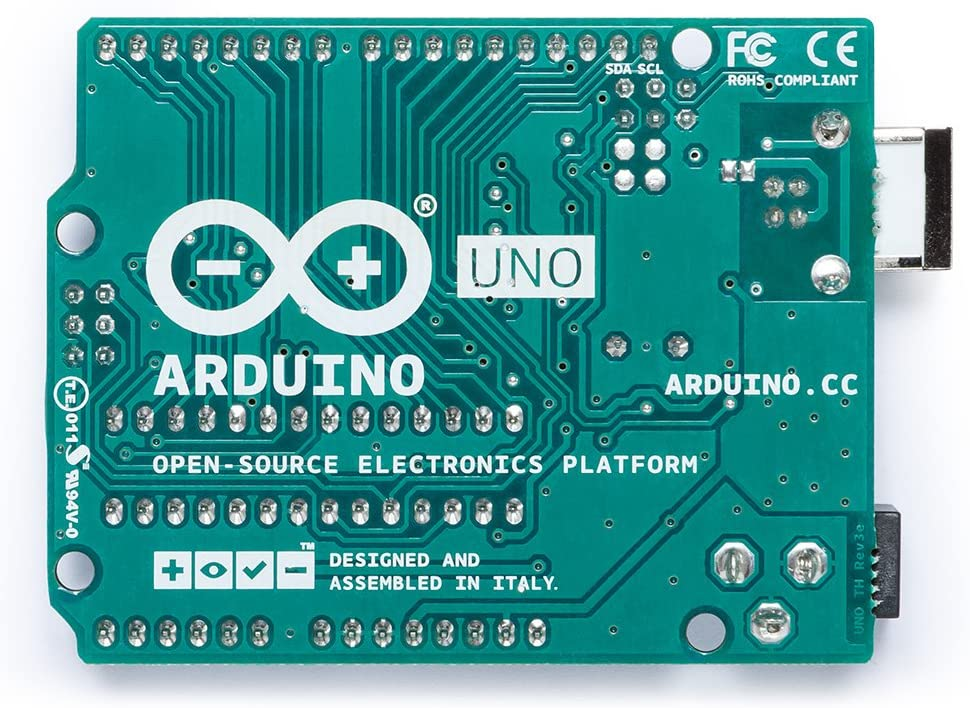

Supplement: Supplementary file 1 [file ijerph-18-13190-s001.zip › Suppl Figure S1.tif]

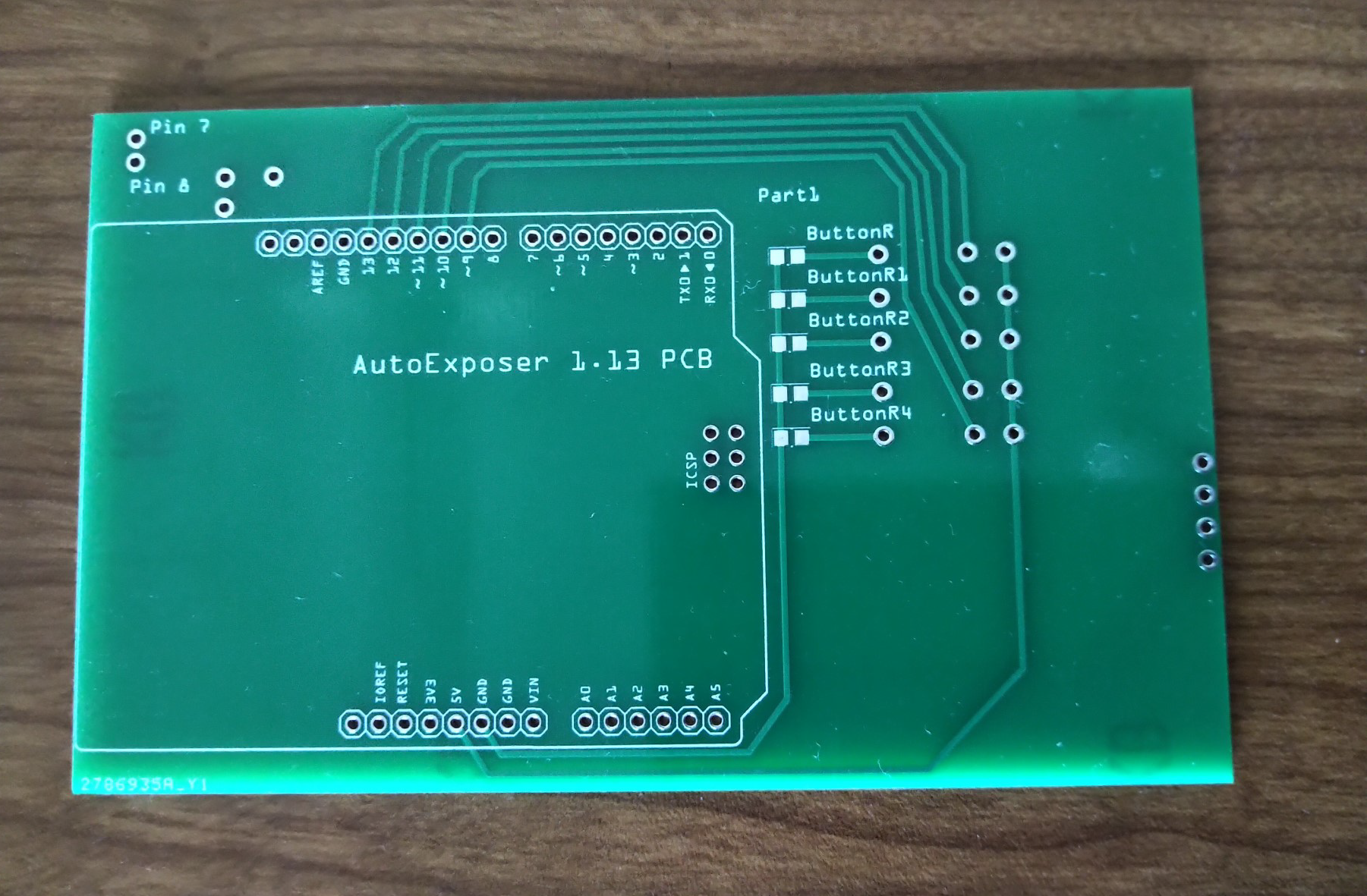

Supplement: Supplementary file 1 [file ijerph-18-13190-s001.zip › Suppl Figure S2.tif]

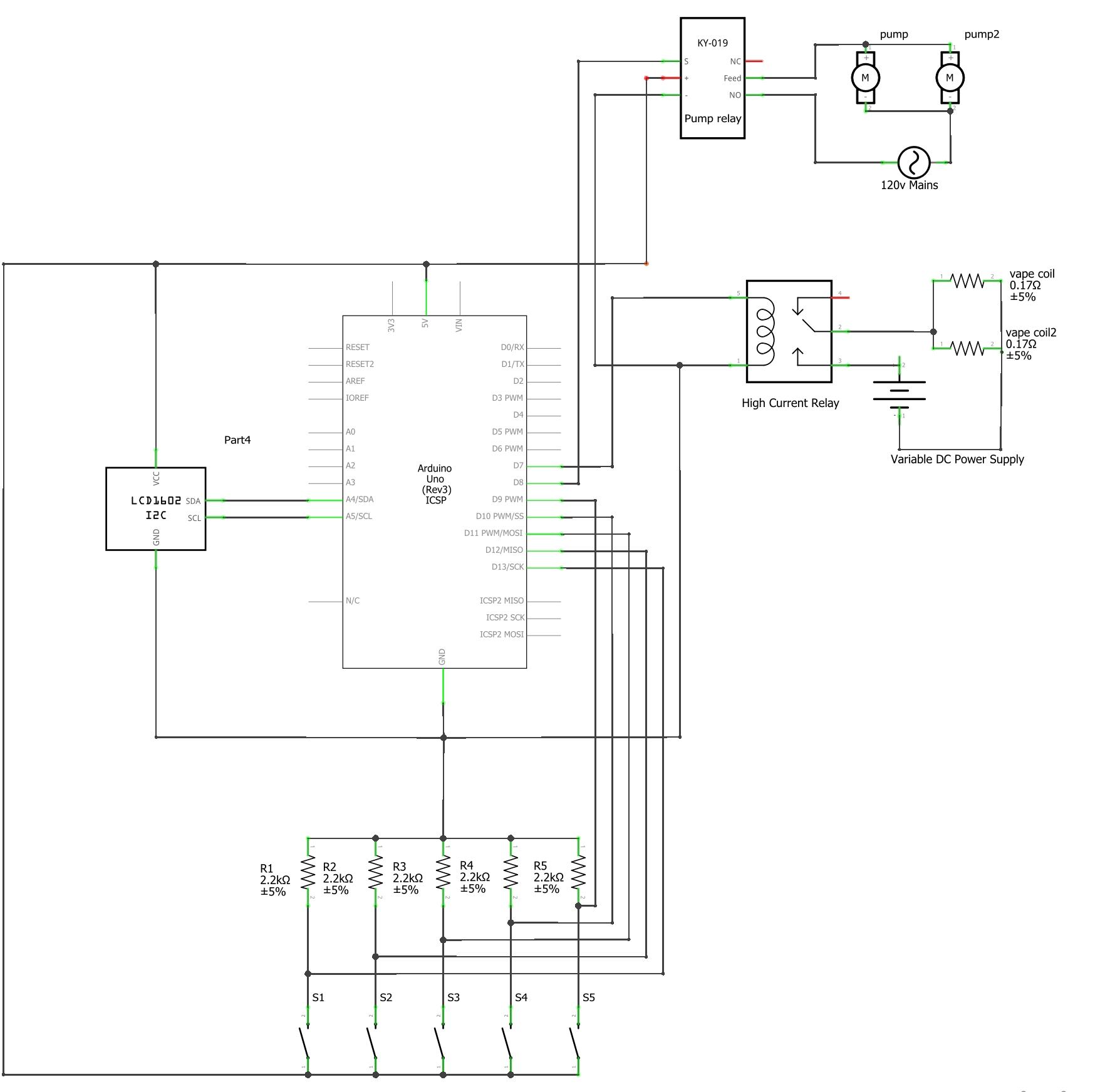

Supplement: Supplementary file 1 [file ijerph-18-13190-s001.zip › Suppl Figure S3.tif]

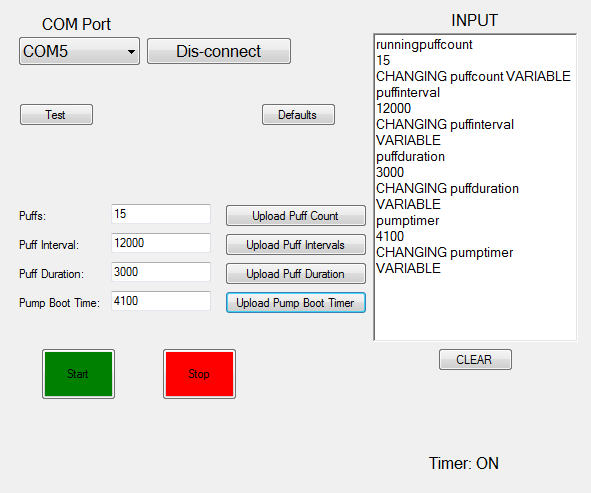

Supplement: Supplementary file 1 [file ijerph-18-13190-s001.zip › Suppl Figure S4.tif]
